# Short-term efficacy of biologics targeting the IL-17/IL-23 axis in scalp, nail, and palmoplantar psoriasis: a network meta-analysis of randomized controlled trials

**DOI:** 10.3389/fimmu.2026.1846290

**Published:** 2026-07-22

**Authors:** Shouxu Zhang, TszLeong Lam, Yue Du, Xiaoxuan Chen, Haomin Zhang, Lingling Li, Guangzhong Zhang

**Affiliations:** 1 Department of Dermatology, Dongzhimen Hospital, Beijing University of Chinese Medicine, Beijing, China; 2 Graduate School, Beijing University of Chinese Medicine, Beijing, China; 3 Capital Medical University, Beijing, China; 4 Beijing Hospital of Chinese Medicine, Beijing, China

**Keywords:** difficult-to-treat psoriasis, interleukin-17 inhibitors, interleukin-23 inhibitors, nail psoriasis, palmoplantar psoriasis, scalp psoriasis

## Abstract

**Background:**

Scalp, nail, and palmoplantar psoriasis are termed “difficult-to-treat sites” owing to their unique anatomical features and therapeutic resistance, substantially impairing patient quality of life. Although anti-IL-17 and anti-IL-23 biologics are widely used, head-to-head comparative evidence for achieving high-level lesion clearance at these specific sites remains limited.

**Methods:**

Following PRISMA-NMA guidelines, we systematically searched PubMed, Embase, and other databases from inception through November 2025 for randomized controlled trials (RCTs). Primary outcomes were defined as complete or near-complete clearance. Frequentist network meta-analysis was performed to calculate odds ratios (ORs), with treatment rankings derived from surface under the cumulative ranking curve (SUCRA) values.

**Results:**

Twenty-four RCTs involving 5,946 patients and eight biologics plus placebo were included. For palmoplantar psoriasis, secukinumab ranked highest (SUCRA = 79.7%), followed by bimekizumab (72.2%) and ustekinumab (69.7%). For nail psoriasis, bimekizumab ranked first (78.9%), followed by ixekizumab (77.2%) and brodalumab (67.5%); however, local inconsistency for the ixekizumab–placebo comparison and low-certainty evidence warrant caution. For scalp psoriasis, brodalumab (87.7%) and ixekizumab (86.9%) ranked highest, followed by bimekizumab (69.0%) and guselkumab (65.1%); the brodalumab estimate relied on a single contributing study.

**Conclusion:**

During induction-phase follow-up, IL-17 inhibitors tended to rank highly for complete or near-complete clearance at difficult-to-treat psoriasis sites. Bimekizumab and ixekizumab showed consistently favorable rankings across sites, but treatment selection should consider certainty of evidence, sensitivity analyses, long-term response, and patient-level factors.

**Systematic review registration:**

https://www.crd.york.ac.uk/PROSPERO, identifier CRD420251271255.

## 1 Introduction

Psoriasis is a chronic immune-mediated inflammatory skin disease affecting 2% to 4% of the global population (1). Beyond the characteristic plaques on the trunk and limbs, some patients experience predominant involvement of specific anatomical sites that constitute their primary clinical concern. These locations—the scalp, nails, and palmoplantar regions—possess unique anatomical features that complicate treatment delivery, contribute to high recurrence rates, and substantially affect appearance or function. They are commonly designated “difficult-to-treat areas” or “high-impact regions” (2). Because lesions at these sites are highly visible or functionally critical, they severely impair patients’ appearance and quality of life while imposing considerable psychosocial burden (3–5). Achieving high-level clearance in these areas therefore carries important clinical significance beyond controlling overall disease activity.

Conventional topical regimens frequently yield suboptimal responses at these sites (3, 6). Scalp hair impedes uniform application of topical agents and limits phototherapy penetration; the compact architecture of the nail unit restricts drug access to the nail bed and matrix; and the thickened stratum corneum of palmoplantar skin—compounded by friction and weight-bearing forces—reduces drug absorption and increases recurrence risk. Topical treatments also require frequent application, exhibit relatively slow onset, and create practical inconveniences such as greasiness and residue, resulting in poor adherence and compromised efficacy (7–9). When difficult-to-treat sites are severely affected or substantially impair quality of life, topical monotherapy often fails to achieve treatment goals, necessitating systemic approaches including biologics (10).

The interleukin-23 (IL-23)/interleukin-17 (IL-17) axis represents a central pathway driving psoriatic inflammation (11). IL-23 stimulates proliferation and activation of T helper 17 (Th17) cells, which secrete proinflammatory cytokines including IL-17A and IL-17F, triggering keratinocyte hyperproliferation and inflammatory cascades (12). Biologics targeting this pathway—including the IL-17 inhibitors secukinumab, ixekizumab, brodalumab, and bimekizumab, as well as the IL-23 inhibitors guselkumab and risankizumab—have achieved substantial progress in treating moderate-to-severe plaque psoriasis, enabling high levels of skin clearance (13). Earlier studies demonstrate that these agents produce clinically meaningful improvements at specialized sites including the scalp, nails, and palmoplantar areas (14, 15).

Despite widespread clinical adoption of biologics, optimal treatment strategies for difficult-to-treat psoriasis remain contested. Existing randomized controlled trials (RCTs) have predominantly used overall skin clearance measures such as Psoriasis Area and Severity Index (PASI) 75/90 as primary endpoints, with few head-to-head studies focused on specific anatomical sites (16). Different agents may exhibit distinct efficacy profiles across the scalp, nails, and palmoplantar regions, leaving clinicians with limited evidence when managing patients whose disease predominantly involves a particular site. This study conducts a network meta-analysis (NMA) systematically incorporating relevant RCTs, using site-specific clearance or near-clearance outcomes to compare and rank the efficacy of anti-IL-17 and anti-IL-23 biologics in treating scalp, nail, and palmoplantar psoriasis, providing an evidence base for precision treatment decisions in clinical practice.

## 2 Materials and methods

**Figure 1:**
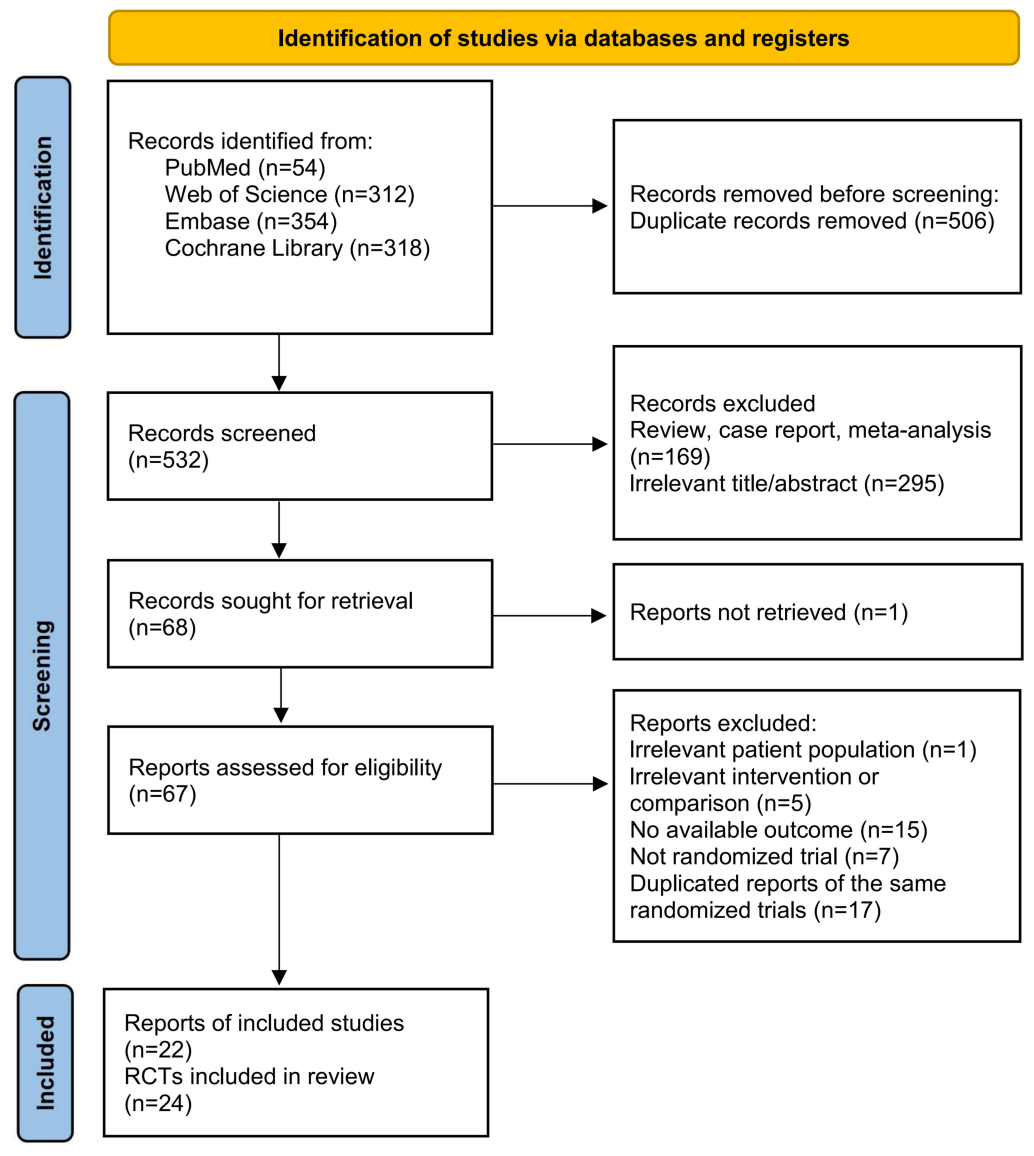
PRISMA flow diagram of the literature search and selection process. Twenty-two reports encompassing 24 randomized controlled trials were included in the network meta-analysis.

This NMA was conducted according to the Preferred Reporting Items for Systematic Reviews and Meta-Analyses extension statement for network meta-analyses (17) (PRISMA-NMA) (**Figure 1**). Although several head-to-head psoriasis RCTs comparing biologics in the IL-17/23 pathway have been published, eligible trials with extractable site-specific complete or near-complete clearance outcomes were limited and did not connect all interventions of interest. We therefore performed NMA to compare these agents and generate probabilistic efficacy rankings (18). The study protocol was registered with the Prospective Register of Systematic Reviews (CRD420251271255) to ensure transparency, reliability, and novelty.

### 2.1 Data sources and search strategy

We systematically searched PubMed, EMBASE, the Cochrane Library, and Web of Science from database inception through November 1, 2025. Search terms included “psoriasis”, “palmoplantar”, “hands”, “feet”, “nail”, “scalp”, “Interleukin-23/antagonists and inhibitors”, “Interleukin-17/antagonists and inhibitors”, “secukinumab”, “ixekizumab”, “bimekizumab”, “brodalumab”, “ustekinumab”, “guselkumab”, “tildrakizumab”, “risankizumab”, and “randomized clinical trial” (**Supplementary Table 2**). We combined free-text and controlled vocabulary terms without language restrictions.

### 2.2 Selection criteria

Inclusion criteria: (1) Participants: Adults (≥18 years) with confirmed psoriasis and documented involvement of specific sites—nails, scalp, or palmoplantar areas. (2) Interventions: Monotherapy with biologics targeting the IL-17 or IL-23 pathways—secukinumab, ixekizumab, bimekizumab, brodalumab, ustekinumab, guselkumab, tildrakizumab, or risankizumab—administered at dosages and frequencies consistent with approved labeling or trial protocols. (3) Comparators: Placebo, other biologics, or conventional systemic therapies. (4) Outcomes: Studies were eligible if they reported the number of patients achieving complete or near-complete clearance at the target difficult-to-treat site. Complete or near-complete clearance was defined as follows: hand and foot Physician’s Global Assessment (hf-PGA) 0/1, palmoplantar Investigator’s Global Assessment (ppIGA) 0/1, or Palmoplantar Psoriasis Area and Severity Index (PPASI) 100; nails—Nail Psoriasis Severity Index (NAPSI)=0, modified NAPSI (mNAPSI)=0, or fingernail Physician’s Global Assessment (f-PGA) 0/1; scalp—Psoriasis Scalp Severity Index (PSSI) 100 or scalp-specific Investigator’s Global Assessment/Scalp Physician’s Global Assessment (ss-IGA/ScPGA) 0/1. (5) Study design: Randomized controlled trials.

We harmonized outcomes representing complete or near-complete clearance across different assessment instruments for each anatomical site. This approach is justified by: (1) all selected thresholds represent clinically equivalent states of complete or near-complete disease resolution—specifically, achieving no detectable disease (score=0) or minimal residual disease (score=1 on global assessments, or 100% improvement on area-severity indices) on validated, site-specific assessment instruments that measure the same underlying clinical construct of skin clearance; (2) similar endpoint harmonization strategies pooling ‘clear/almost clear’ outcomes from different validated instruments have been successfully applied in recent network meta-analyses of site-specific psoriasis (19, 20), establishing methodological precedent for this approach; and (3) heterogeneous outcome reporting across trials necessitated this pragmatic pooling to enable indirect treatment comparisons while maintaining clinical validity, relevance to patient-centered outcomes, and alignment with regulatory definitions of high-level treatment response. We conducted all analyses using the intent-to-treat principle with outcomes assessed at 12–16 weeks, the standard induction-phase timepoint.

Exclusion criteria: (1) Non-randomized studies, reviews, case reports, and duplicate publications. (2) Studies that did not report site-specific subgroup data separately or from which required data could not be extracted. (3) Studies primarily examining topical agents or non-mainstream therapies. Two reviewers independently screened RCTs by title and abstract. All included trials underwent dual verification to ensure the data represented the most recently published information.

### 2.3 Data extraction and quality assessment

Three investigators independently screened literature and extracted data following the PRISMA-NMA framework, with a fourth investigator consulted to resolve disagreements. Extracted information included study characteristics (authors, year, National Clinical Trial [NCT] number), methodological features, baseline participant characteristics (sample size, age, sex, disease duration, prior biologic exposure, race), interventions, follow-up duration, and outcome data (total number in each group and number of events achieving complete clearance).

Methodological quality was assessed using the Cochrane Risk of Bias 2.0 (RoB 2.0) tool (21). We defined the unit of assessment as the specific outcome measure for each difficult-to-treat site—nails, scalp, or palmoplantar areas—rather than the trial or publication as a whole. For pooled or subgroup analyses derived from the same parent trials (e.g., the UNCOVER and AMAGINE series) but reporting outcomes for different sites, we conducted separate assessments for each site-specific outcome. Risk of bias was evaluated across five domains: randomization process, deviations from intended interventions, missing outcome data, measurement of the outcome, and selection of the reported result. Each domain was classified as low risk, high risk, or having some concerns.

### 2.4 Statistical analysis

Network meta-analysis was performed using a frequentist framework with the mvmeta and network packages in Stata 17.0 MP. Given the dichotomous nature of outcomes, we used odds ratios (ORs) with 95% confidence intervals (CIs) as the effect measure. In network plots, node size reflected total sample size for each intervention, while edge thickness represented the number of RCTs contributing to each pairwise comparison. For open-loop network structures, we applied a consistency model directly. When closed loops were present, we first conducted a global inconsistency test; *P* > 0.05 indicated satisfaction of the consistency assumption, permitting use of the consistency model. Additionally, we employed node-splitting to examine local inconsistency and loop-specific inconsistency factors to assess consistency within closed loops. When the 95% CI of the inconsistency factor included zero, this indicated acceptable agreement between direct and indirect evidence; otherwise, we explored sources of inconsistency. Intervention rankings were derived from surface under the cumulative ranking curve (SUCRA) values and displayed as cumulative probability plots. Publication bias and small-study effects were assessed using comparison-adjusted funnel plots.

To verify robustness of primary findings, we conducted leave-one-out sensitivity analyses. In each iteration, a single study was excluded and the network reconstructed from remaining data, with effect estimates relative to control recalculated under the consistency assumption. Results were deemed robust if effect direction remained unchanged and magnitude varied within reasonable bounds after removal of any individual study. To clarify potential covariate effects, we performed univariate network meta-regression. In these models, candidate covariates were entered as sole predictors, with relative treatment effects versus control as the dependent variable, using random-effects structure and restricted maximum likelihood estimation for between-study variance (τ²). We reported regression coefficients, 95% CIs, and *P*-values from Wald tests; *P* < 0.05 indicated significant effect modification by the covariate.

### 2.5 GRADE assessment

Evidence certainty was graded using the Grading of Recommendations Assessment, Development and Evaluation (GRADE) framework implemented through the Confidence in Network Meta-analysis (CINeMA) tool, with RCTs initially assigned high certainty. Within-study bias was assessed using RoB 2.0 for individual trials, then aggregated to the comparison level via CINeMA’s contribution matrix. Indirectness was evaluated based on transitivity assumptions, examining consistency of population, intervention, and outcome characteristics across direct and indirect comparisons. Before accepting indirect comparisons, we compared potential effect modifiers across treatment nodes, including prior biologic exposure, disease duration, baseline PASI, race, and follow-up duration. We considered transitivity plausible when these factors showed no major systematic imbalance that would make one treatment node clinically incomparable with another; residual imbalances were addressed through meta-regression where data permitted and through CINeMA indirectness judgments. Imprecision was judged by whether 95% CIs crossed predefined minimum important difference (MID) thresholds or the null. Heterogeneity was assessed by comparing confidence and prediction intervals relative to the MID, supplemented by τ² and I² statistics. When closed loops existed, incoherence was evaluated using CINeMA’s built-in direct-indirect consistency tests—Separating Indirect from Direct Evidence (SIDE)/node-splitting or design-by-treatment methods, to determine compatibility. Publication bias incorporated consideration of trial registry searches and grey literature, alongside small-study effects examined via comparison-adjusted funnel plots and regression tests. Each domain was rated as having no concerns, some concerns, or major concerns, with certainty downgraded one level for each “some concerns” rating and two levels for “major concerns”, yielding final evidence certainty classifications of high, moderate, low, or very low.

## 3 Results

### 3.1 Systematic review and characteristics of the included studies

The initial literature search identified 1,038 records. After removal of 506 duplicates, 532 records were screened, of which 464 were excluded at the title and abstract stage. Sixty-eight reports were sought for retrieval, one was not retrieved, and 67 reports were assessed for eligibility. Ultimately, 22 publications (7, 15, 22–41) encompassing 24 RCTs were included (**Figure 1**), representing 5,946 patients with psoriasis. In subgroup analyses focusing on difficult-to-treat sites, 4,580 patients had nail psoriasis, 4,565 had scalp psoriasis, and 1,323 had palmoplantar psoriasis.

The included studies were published between 2016 and 2025. Interventions comprised eight biologics, with placebo as the primary comparator. These agents were categorized according to their molecular targets as follows: IL-17A inhibitors, secukinumab and ixekizumab; dual IL-17A/F inhibitor, bimekizumab; IL-17 receptor A (IL-17RA) inhibitor, brodalumab; IL-23p19 inhibitors, guselkumab, risankizumab, and tildrakizumab; and IL-12/23p40 inhibitor, ustekinumab (**Table 1**). Mean patient age ranged predominantly from 40 to 55 years, with males comprising more than 50% of participants in most studies (range: 45.9%–79.5%). Most studies enrolled predominantly White populations (>70% in most trials), although some included exclusively Asian cohorts (e.g., Ohtsuki et al., Li et al.). We systematically examined key potential effect modifiers across included trials: mean disease duration ranged from 7.1 to 20.5 years, prior biologic exposure from 0% to 46.5%, and baseline PASI from 6.1 to 26.3 (**Table 2**). Detailed study characteristics and baseline data are presented in **Tables 1**, **2**.

**Table 1:** Characteristics of included studies.

Study	Registration no.	Target area	Total sample size (N)	Treatment groups [mechanism] (n)	Timepoint (wks)	Reported outcomes
Menter et al., 2017 (22); Papp et al., 2018 (23); Reich et al., 2017 (24) (UNCOVER-1, -2, -3)	NCT01474512 NCT01597245 NCT01646177	Palmoplantar, Nail,Scalp	1961	Ixekizumab [IL-17A] (1169) Placebo (792)	12	PPASI 100,NAPSI = 0, PSSI 100
Gottlieb et al., 2017 (25)	NCT01806597	Palmoplantar	137	Secukinumab [IL-17A] (69) Placebo (68)	16	ppIGA 0/1
Lebwohl et al., 2024 (26)	NCT04713592	Palmoplantar	174	Risankizumab [IL-23p19] (87) Placebo (87)	16	ppIGA 0/1
Passeron et al., 2023 (27)	NCT03998683	Palmoplantar	117	Guselkumab [IL-23p19] (78) Placebo (39)	16	ppIGA 0/1
Gordon et al., 2021 (28)	NCT03410992	Palmoplantar, Nail, Scalp	435	Bimekizumab [IL-17A/F] (349) Placebo (86)	16	pp-IGA 0/1, ss-IGA, mNAPSI 100
Reich et al., 2021 (29)	NCT03370133	Palmoplantar, Nail, Scalp	567	Bimekizumab [IL-17A/F] (321) Ustekinumab [IL-12/23p40] (163) Placebo (83)	16	pp-IGA 0/1, ss-IGA, mNAPSI 100
Reich et al., 2016 (42)	NCT02207244	Palmoplantar, Nail, Scalp	744	Guselkumab [IL-23p19] (496) Placebo (248)	16	ss-IGA 0/1,f-PGA 0/1, hf-PGA 0/1
Blauvelt et al., 2017 (15)	NCT02207231	Palmoplantar, Nail, Scalp	503	Guselkumab [IL-23p19] (329) Placebo (174)	16	ss-IGA 0/1,f-PGA 0/1, hf-PGA 0/1
Li et al., 2025 (43)	NCT03364309	Palmoplantar, Scalp	264	Ixekizumab [IL-17A] (176) Placebo (88)	12	PSSI 100, PPASI 100
Elewski et al., 2022 (7) (AMAGINE-1)	NCT01708590	Scalp	442	Brodalumab [IL-17RA] (222) Placebo (220)	12	PSSI 100
Elewski et al., 2022 (7) (AMAGINE-2/-3)	NCT01708603/NCT01708629	Nail	2473	Brodalumab [IL-17RA] (1236) Ustekinumab [IL-12/23p40] (613) Placebo (624)	52	NAPSI=0
Mease et al., 2017 (31)	NCT01695239	Nail	209	Ixekizumab [IL-17A] (103) Placebo (106)	24	NAPSI=0
Wasel et al., 2020 (32)	NCT02561806	Nail	302	Ixekizumab [IL-17A] (166) Ustekinumab [IL-12/23p40] (136)	52	NAPSI=0
Coates et al., 2025 (33)	NCT03675308	Nail	964	Risankizumab [IL-23p19] (483) Placebo (481)	24	f-PGA 0/1
Blauvelt et al., 2021 (34)	NCT03573323	Nail	1027	Ixekizumab [IL-17A] (520) Guselkumab [IL-23p19] (507)	12	f-PGA 0/1
Bagel et al., 2017 (35)	NCT02267135	Scalp	102	Secukinumab [IL-17A] (51) Placebo (51)	12	ss-IGA 0/1
Stein Gold et al., 2025 (36)	NCT06039189	Scalp	338	Guselkumab [IL-23p19] (225) Placebo (113)	16	ss-IGA 0/1
Song et al., 2025 (37)	NCT05969223	Scalp	105	Risankizumab [IL-23p19] (51) Placebo (54)	16	ss-IGA 0/1
Ohtsuki et al., 2018 (38)	NCT02325219	Scalp	127	Guselkumab [IL-23p19] (63) Placebo (64)	16	ss-IGA 0/1
McMichael A et al., 2025 (39)	NCT05272150	Scalp	102	Guselkumab [IL-23p19] (76) Placebo (26)	16	ss-IGA 0/1
Gebauer et al., 2024 (40)	NCT03897088	Scalp	171	Tildrakizumab [IL-23p19] (89) Placebo (82)	16	ss-IGA 0/1

**Table 2:** Baseline demographics and clinical characteristics.

Study	NCT number	Age (years)	Men(%)	Race (% White)	Disease duration (years)	Baseline PASI	Previous bio therapy (%)
Menter et al., 2017 (22); Papp et al., 2018 (23); Reich et al., 2017 (24)	NCT01474512 NCT01597245 NCT01646177	45.8	67.6	92.8	18.9	23.4	29.1
Gottlieb et al., 2017 (25)	NCT01806597	49.8	52.6	96.4	9.8	7.9	9.5
Lebwohl et al., 2024 (26)	NCT04713592	55.4	51.2	85.1	10	11.7	10.9
Passeron et al., 2023 (27)	NCT03998683	51.6	51.3	–	12.6	6.1	0
Gordon et al., 2021 (28)	NCT03410992	44.3	71.8	92.8	19.5	20.3	43.8
Reich et al., 2021 (29)	NCT03370133	46.1	71.4	74.3	17.1	21.5	39.1
Reich et al., 2016 (42)	NCT02207244	43.6	70.2	82.6	17.9	21.8	20.9
Blauvelt et al., 2017 (15)	NCT02207231	44.2	71.3	80.9	17.8	21.5	20.9
Li et al., 2025 (43)	NCT03364309	40.1	78	0	16.4	25.2	9.5
Elewski et al., 2022 (7)	NCT01708590	46.5	73	91.5	20.5	19.6	46.5
Elewski et al., 2022 (7)	NCT01708603/ NCT01708629	45	68.6	90.4	18.9	20.2	27.2
Mease et al., 2017 (31)	NCT01695239	50.2	45.9	93.3	16.5	6.1	0
Wasel et al., 2020 (32)	NCT02561806	43.3	66.8	94.4	18.1	19.8	14.1
Coates et al., 2025 (33)	NCT03675308	51.2	50.4	93.9	7.1	10.5	0
Blauvelt et al., 2021 (34)	NCT03573323	47.8	63.5	84.7	16.9	19.4	26
Bagel et al., 2017 (35)	NCT02267135	41.9	47	80.4	14.9	9.3	0
Stein Gold et al., 2026 (36)	NCT06039189	46.2	51.2	73.8	16.9	9	0
Song et al., 2025 (37)	NCT05969223	46.4	56.2	83.6	15.4	12.4	37.1
Ohtsuki et al., 2018 (38)	NCT02325219	48.1	79.5	0	14	26.3	16.5
McMichael et al., 2025 (39)	NCT05272150	42.4	56.8	0	11.3	14.6	12.7
Gebauer et al., 2024 (40)	NCT03897088	44.8	60.3	78.9	15	18.8	9.3

These baseline and study-level characteristics were also considered when evaluating the plausibility of transitivity across treatment nodes. Overall, the included trials enrolled broadly comparable adult patients with site-specific psoriasis, and most outcomes were assessed during the induction phase at 12–16 weeks. However, potential effect modifiers were not perfectly balanced across all treatment nodes, particularly prior biologic exposure, disease duration, baseline PASI, and racial composition. Accordingly, potential effect modification was examined in the site-specific meta-regression analyses reported below.

Quality assessment using RoB 2.0 (**Supplementary Figure 1**) classified 15 of 22 publications as low risk overall, with 7 rated as having some concerns. To address selective-reporting concerns, we classified each site-specific outcome as a prespecified endpoint, protocol-defined endpoint, *post hoc* subgroup analysis, or *post hoc* regional analysis with prospectively collected outcomes (**Supplementary Table 3**). Some concerns in the selection of the reported result domain were mainly assigned to *post hoc* subgroup analyses from broader parent RCTs.

### 3.2 Palmoplantar psoriasis

#### 3.2.1 Network meta-analysis and efficacy

The network structure for palmoplantar outcomes formed closed loops, prompting a global inconsistency test that yielded *P* = 0.5054, well above the 0.05 threshold, indicating no rejection of the consistency assumption. Node-splitting analysis for local inconsistency likewise produced *P* > 0.05 for all comparison nodes (**Supplementary Table 4**). Loop inconsistency analysis examining agreement between direct and indirect evidence showed that confidence intervals for all inconsistency factors crossed zero, confirming satisfactory overall network consistency (**Supplementary Figure 2**).

**Figure 2:**
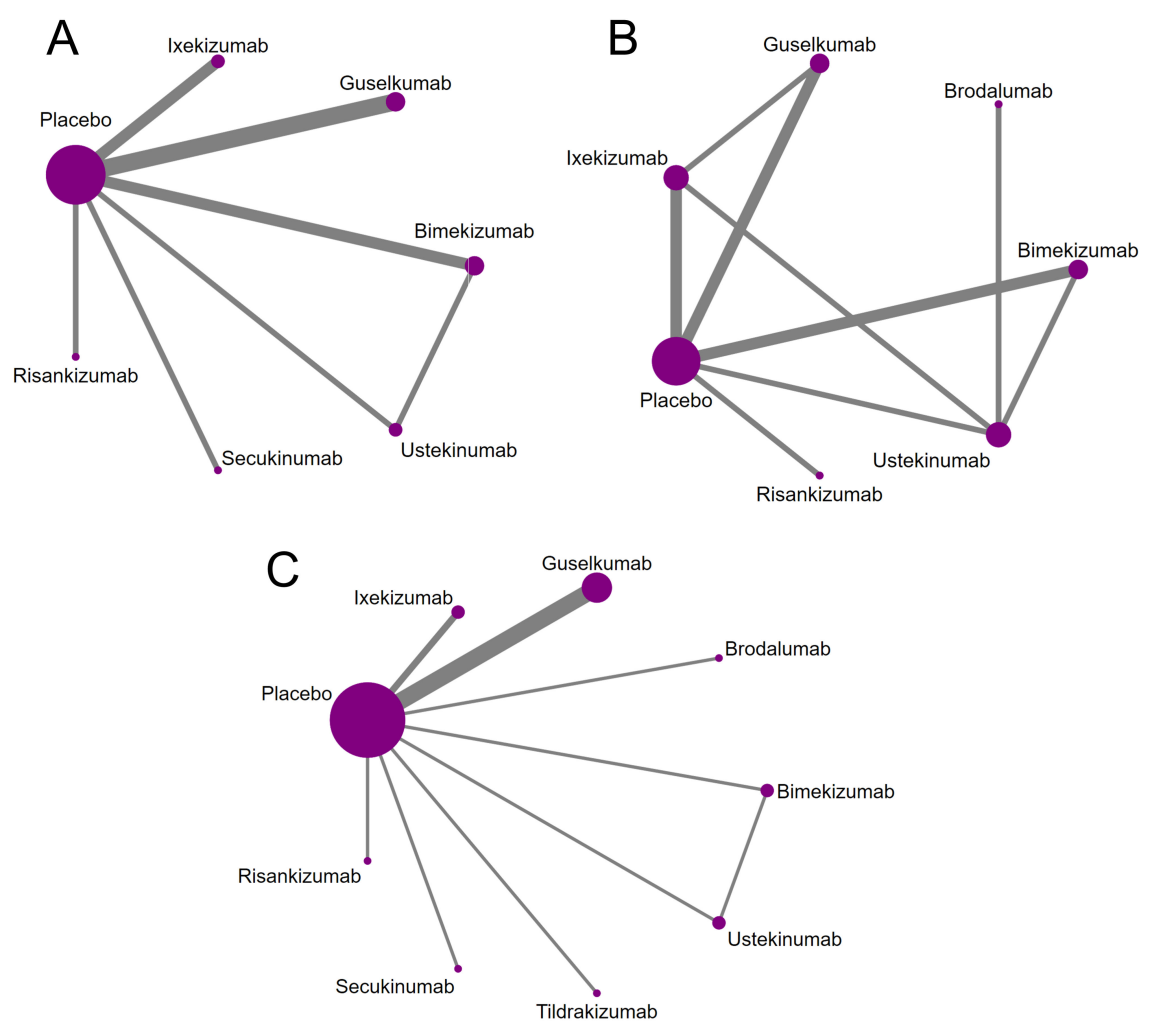
Network plots for complete or near-complete site-specific clearance. **(A)** Palmoplantar; **(B)** Nail; **(C)** Scalp. The size of the nodes is proportional to the number of patients (or trials) assigned to each intervention. The lines between nodes represent direct comparisons between interventions, with the thickness of the lines proportional to the number of studies (or sample size) involving the comparison.

**Figure 3:**
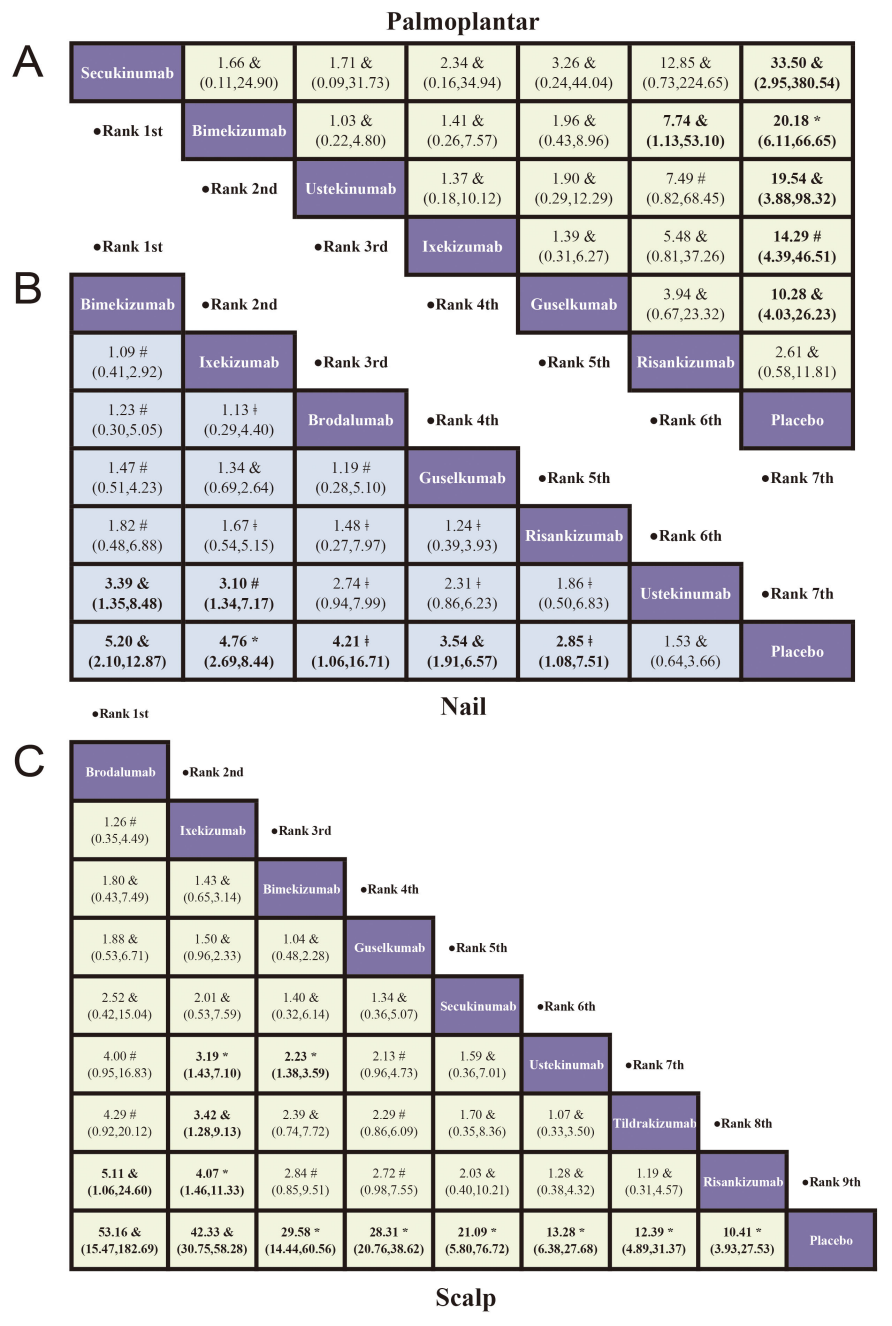
League tables of comparative efficacy for complete or near-complete clearance. **(A)** palmoplantar, **(B)** nail, and **(C)** scalp psoriasis. Data are presented as odds ratios (ORs) with 95% confidence intervals (CIs). Data are presented as odds ratios (ORs) with 95% confidence intervals (CIs), with each cell comparing the higher-ranked treatment with the lower-ranked treatment. Bold text indicates statistically significant results (95% CI excluding 1). Certainty of evidence was assessed using the CINeMA tool based on the GRADE framework and is denoted as follows: *High; ^&^Moderate; ^#^Low; ^‡^Very low.

Nine RCTs investigating palmoplantar psoriasis were included, covering six active biologics plus placebo (**Figure 2A**). One study provided direct head-to-head evidence between active biologics, namely bimekizumab versus ustekinumab, whereas the remaining comparisons were placebo-controlled. The efficacy endpoint was defined as the proportion of patients achieving complete or near-complete clearance at weeks 12–16, specifically hf-PGA 0/1, ppIGA 0/1, or PPASI 100. All biologics except risankizumab demonstrated superior efficacy compared with placebo. The largest effect sizes were observed for secukinumab (OR = 33.50, 95% CI: 2.95–380.54) and bimekizumab (OR = 20.18, 95% CI: 6.11–66.65). Ustekinumab (OR = 19.54, 95% CI: 3.88–98.32) and guselkumab (OR = 10.28, 95% CI: 4.03–26.23) also showed statistically significant improvements (**Figure 3A**; **Table 3**).

**Table 3:** Summary of treatment rankings, effect estimates, and evidence certainty for complete or near-complete clearance by anatomical site.

Site	Rank	Treatment	SUCRA (%)	OR vs placebo (95% CI)	Certainty
Palmoplantar	1	Secukinumab	79.7	33.50 (2.95, 380.54)	Moderate
	2	Bimekizumab	72.2	20.18 (6.11, 66.65)	High
	3	Ustekinumab	69.7	19.54 (3.88, 98.32)	Moderate
	4	Ixekizumab	59.7	14.29 (4.39, 46.51)	Low
	5	Guselkumab	48.3	10.28 (4.03, 26.23)	Moderate
	6	Risankizumab	18.5	2.61 (0.58, 11.81)	Moderate
Nail	1	Bimekizumab	78.9	5.20 (2.10, 12.87)	Moderate
	2	Ixekizumab	77.2	4.76 (2.69, 8.44)	High
	3	Brodalumab	67.5	4.21 (1.06, 16.71)	Very low
	4	Guselkumab	57.0	3.54 (1.91, 6.57)	Moderate
	5	Risankizumab	47.9	2.85 (1.08, 7.51)	Very low
	6	Ustekinumab	18.3	1.53 (0.64, 3.66)	Moderate
Scalp	1	Brodalumab	87.7	53.16 (15.47, 182.69)	Moderate
	2	Ixekizumab	86.9	42.33 (30.75, 58.28)	Moderate
	3	Bimekizumab	69.0	29.58 (14.44, 60.56)	High
	4	Guselkumab	65.1	28.31 (20.76, 38.62)	High
	5	Secukinumab	52.9	21.09 (5.80, 76.72)	High
	6	Ustekinumab	32.0	13.28 (6.38, 27.68)	High
	7	Tildrakizumab	30.9	12.39 (4.89, 31.37)	High
	8	Risankizumab	25.5	10.41 (3.93, 27.53)	High

Treatments are ordered by SUCRA ranking within each anatomical site. ORs are shown versus placebo. Certainty ratings were based on the CINeMA/GRADE framework. CI, confidence interval; OR, odds ratio; SUCRA, surface under the cumulative ranking curve.

#### 3.2.2 SUCRA ranking

**Figure 4:**
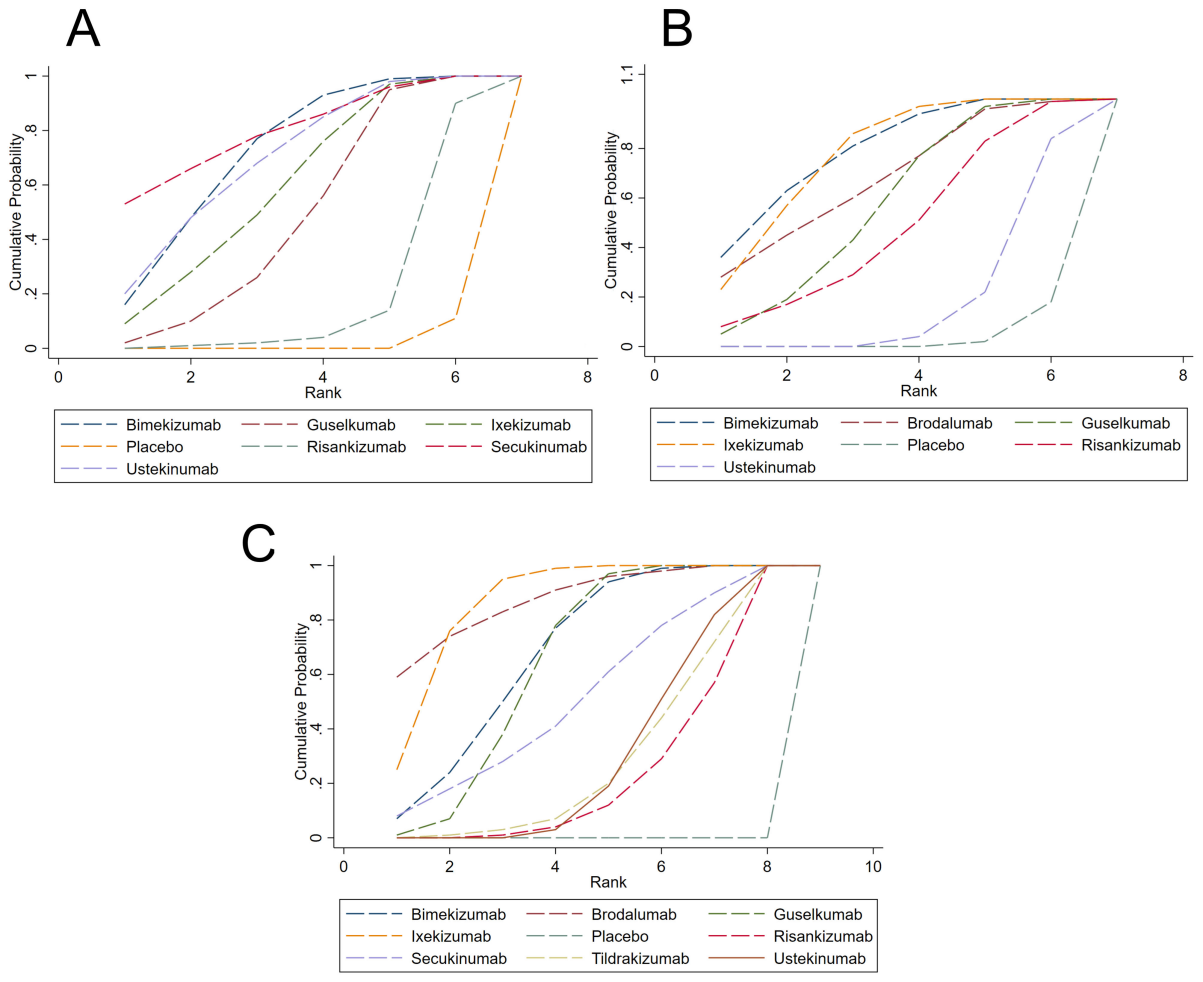
SUCRA plots for complete or near-complete site-specific clearance. **(A)** Palmoplantar; **(B)** nail; **(C)** scalp. Curves closer to the upper-left corner indicate higher cumulative ranking probabilities and more favorable short-term efficacy. SUCRA, surface under the cumulative ranking curve.

Ranking analysis based on SUCRA values showed secukinumab had the highest probability of being the best treatment for palmoplantar psoriasis (SUCRA = 79.7%), followed by bimekizumab (SUCRA = 72.2%), ustekinumab (SUCRA = 69.7%), and ixekizumab (SUCRA = 59.7%) (**Figure 4A**; **Supplementary Tables 5**, **6**).

#### 3.2.3 GRADE assessment

Assessment of evidence certainty using the CINeMA framework revealed variability across comparisons, ranging from high to low. For palmoplantar clearance, 1 of 21 comparisons (5%) received high certainty, 18 (86%) received moderate certainty, and 2 (10%) received low certainty (**Supplementary Table 7**).

#### 3.2.4 Sensitivity analysis, meta-regression, and publication bias

Leave-one-out sensitivity analysis revealed no single study exerting decisive influence on network effect estimates. After sequentially excluding each study and reanalyzing the remaining data under a consistency random-effects model, all major biologics maintained effect directions favoring treatment over placebo (all ORs >1), with minimal fluctuation in effect magnitude, substantial overlap of confidence intervals, and no meaningful changes in statistical significance, confirming result stability (**Supplementary Table 8**).

For palmoplantar outcomes, we performed univariate network meta-regression using mean patient age, proportion with prior biologic exposure, mean disease duration, baseline PASI score, and study follow-up duration as covariates to test whether these factors modified relative treatment effects versus placebo (41). None of these covariates significantly influenced treatment effects (**Supplementary Tables 9**–**13**).

**Figure 5:**
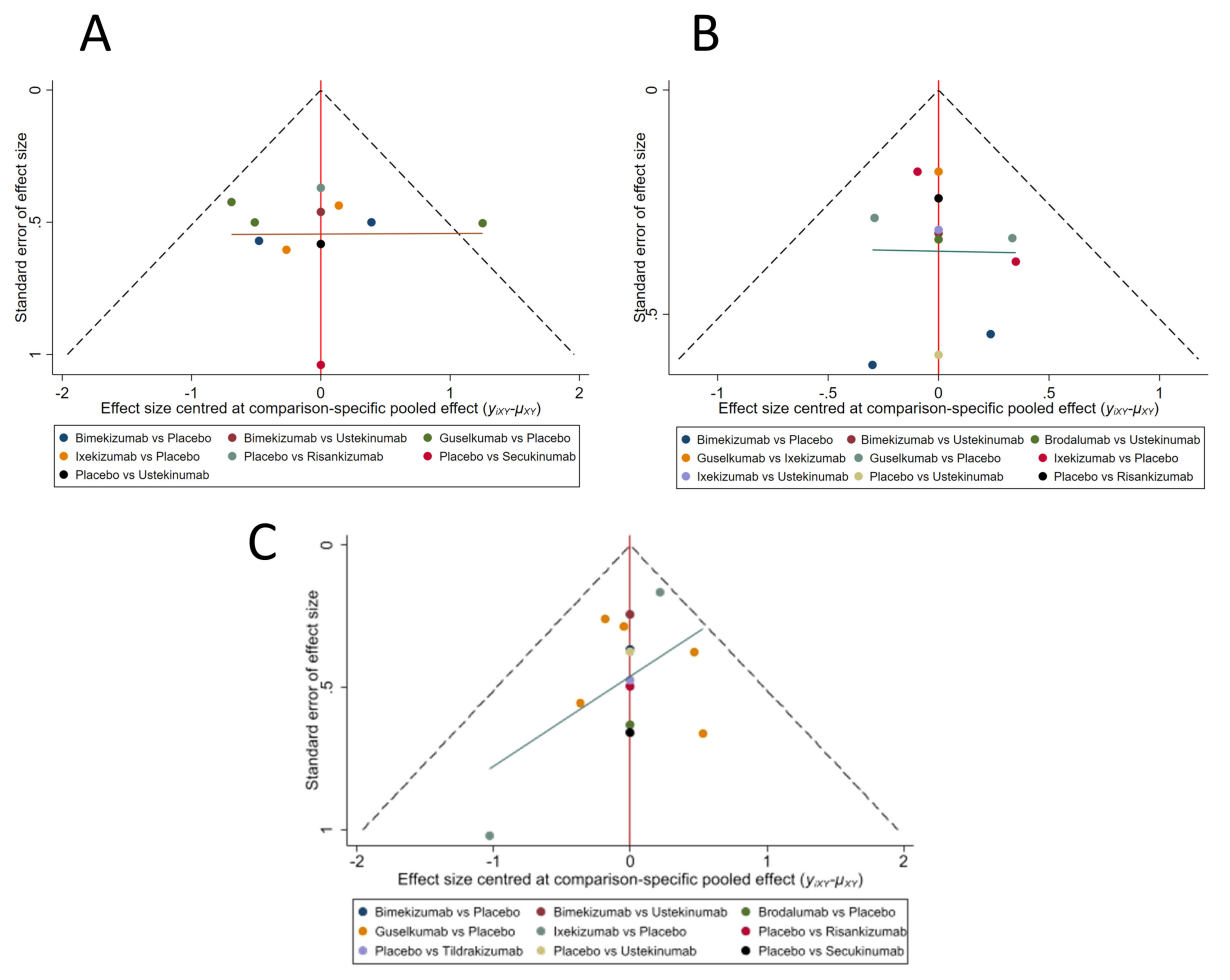
Comparison-adjusted funnel plots for publication bias. **(A)** Palmoplantar psoriasis; **(B)** nail psoriasis; **(C)** scalp psoriasis. A broadly symmetrical distribution suggests no obvious evidence of small-study effects or publication bias.

The funnel plot (**Figure 5A**) showed roughly symmetrical distribution of study points with no obvious outliers, and the regression line representing Egger’s test was approximately horizontal, suggesting low likelihood of publication bias.

### 3.3 Nail psoriasis

#### 3.3.1 Network consistency

The network structure for nail outcomes formed closed loops, necessitating a global inconsistency test that yielded *P* = 0.082, above the 0.05 threshold, indicating no rejection of the consistency assumption. Node-splitting analysis for local inconsistency revealed that the comparison between ixekizumab and placebo produced *P* = 0.001, warranting caution in interpretation, while all other comparison nodes showed *P* > 0.05 (**Supplementary Table 14**). Loop inconsistency analysis examining agreement between direct and indirect evidence showed confidence intervals for all inconsistency factors crossing zero, confirming satisfactory overall network consistency (**Supplementary Figure 3**).

#### 3.3.2 Network meta-analysis and efficacy

Ten RCTs examining nail psoriasis were included, covering seven interventions including placebo (**Figure 2B**). Among these, five RCTs provided direct active-comparator evidence, corresponding to four head-to-head biologic comparisons: bimekizumab versus ustekinumab, brodalumab versus ustekinumab, ixekizumab versus ustekinumab, and ixekizumab versus guselkumab. The remaining direct evidence was placebo-controlled. Compared with placebo, bimekizumab (OR = 5.20, 95% CI: 2.10–12.87), ixekizumab (OR = 4.76, 95% CI: 2.69–8.44), brodalumab (OR = 4.21, 95% CI: 1.06–16.71), guselkumab (OR = 3.54, 95% CI: 1.91–6.57), and risankizumab (OR = 2.85, 95% CI: 1.08–7.51) all demonstrated significant improvement in nail disease. Ustekinumab (OR = 1.53, 95% CI: 0.64–3.66), while numerically favoring treatment, did not reach statistical significance. In comparisons between agents, estimates favored bimekizumab (OR = 3.39) and ixekizumab (OR = 3.10) over ustekinumab, but these head-to-head inferences should be interpreted in light of local inconsistency for the ixekizumab-placebo comparison (**Figure 3B**; **Table 3**).

#### 3.3.3 SUCRA ranking

SUCRA rankings placed bimekizumab first (SUCRA = 78.9%), followed by ixekizumab (77.2%) and brodalumab (67.5%) (**Figure 4B**; **Supplementary Tables 5**, **6**).

#### 3.3.4 GRADE assessment

For nail psoriasis, 1 of 21 comparisons (5%) received high certainty, 5 (24%) received moderate certainty, 6 (29%) received low certainty, and 9 (43%) received very low certainty (**Supplementary Table 15**).

#### 3.3.5 Sensitivity analysis, meta-regression, and publication bias

Leave-one-out sensitivity analysis confirmed robust efficacy conclusions for bimekizumab, ixekizumab, and guselkumab. However, brodalumab showed superiority over placebo in the main analysis (OR = 4.21, 95% CI: 1.06–16.71), but after excluding NCT03370133, the 95% CI widened to 0.50–16.95, crossing the null. Similarly, exclusion of NCT02207231 or NCT03410992 resulted in loss of statistical significance. Risankizumab demonstrated superiority over placebo in the main analysis (OR = 2.85, 95% CI: 1.08–7.51), but after excluding AMAGINE_2_3, the effect size decreased with the CI becoming 0.64–3.66; exclusion of NCT02207231 or UNCOVER_1_2_3 also eliminated statistical significance (**Supplementary Table 16**).

Univariate network meta-regression was performed using mean patient age, proportion with prior biologic exposure, mean disease duration, baseline PASI score, and study follow-up duration as covariates. Disease duration showed a significant positive linear association with guselkumab efficacy relative to placebo (coefficient=1.22, *P* = 0.045), suggesting that guselkumab demonstrated relatively better efficacy in studies enrolling patients with longer disease duration. No significant effect modification by disease duration was observed for other biologics. None of the remaining covariates showed statistically significant associations with treatment effects for any intervention versus placebo (**Supplementary Tables 17**–**21**).

The funnel plot (**Figure 5B**) showed study points distributed symmetrically around the centerline (X = 0), with most studies falling within the 95% confidence bounds of the inverted funnel and no obvious outliers, suggesting low likelihood of publication bias.

### 3.4 Scalp psoriasis

#### 3.4.1 Network meta-analysis and efficacy

The network meta-analysis for scalp psoriasis included 12 RCTs covering eight biologics and placebo (**Figure 2C**). The evidence network was predominantly placebo-controlled. Only one study provided direct active-comparator evidence, which compared bimekizumab with ustekinumab. All biologics significantly outperformed placebo in achieving complete or near-complete clearance of scalp lesions (**Figure 3C**; **Table 3**). Brodalumab demonstrated the largest effect estimate (OR = 53.16, 95% CI: 15.47–182.69), followed by ixekizumab (OR = 42.33, 95% CI: 30.75–58.28), bimekizumab (OR = 29.58, 95% CI: 14.44–60.56) and guselkumab (OR = 28.31, 95% CI: 20.76–38.62).

#### 3.4.2 SUCRA ranking

SUCRA rankings placed brodalumab first (SUCRA = 87.7%), followed closely by ixekizumab (86.9%), bimekizumab (69%), and guselkumab (65.1%). Risankizumab (25.5%) ranked relatively lower for this site (**Figure 4C**; **Supplementary Tables 5**, **6**).

#### 3.4.3 GRADE assessment

For scalp psoriasis (**Supplementary Table 22**), 9 of 36 comparisons (25%) received high certainty, 20 (55.5%) received moderate certainty, and 7 (19.4%) received low certainty.

#### 3.4.4 Sensitivity analysis, meta-regression, and publication bias

Leave-one-out sensitivity analysis showed that the direction of effect favoring biologics over placebo was generally retained across the scalp network (**Supplementary Table 23**). The main efficacy estimates for ixekizumab, bimekizumab, guselkumab, ustekinumab, tildrakizumab, and risankizumab remained directionally consistent across estimable iterations. However, ixekizumab showed variation in effect magnitude; after exclusion of the UNCOVER_1_2_3 dataset, the OR increased to 142.45 with a wide confidence interval, indicating that although the direction of effect was stable, the magnitude of the estimate was influenced by key contributing studies. For brodalumab, removal of NCT01708590 rendered the comparison non-estimable because this trial provided the available direct evidence for brodalumab in the scalp network. Therefore, although the main analysis suggested clear superiority of brodalumab over placebo, this estimate should be interpreted cautiously. Overall, the sensitivity analysis supported the general direction of efficacy favoring biologics over placebo, but also indicated that several scalp psoriasis estimates depended on limited direct evidence.

To explore potential sources of heterogeneity, we conducted univariate network meta-regression for scalp outcomes using mean patient age, proportion with prior biologic exposure, mean disease duration, baseline PASI score, and study follow-up duration as covariates. None of these covariates showed a statistically significant association with relative treatment effects in the available models (**Supplementary Tables 24**–**28**), suggesting no clear evidence of effect modification by these study-level characteristics.

Assessment of publication bias using the comparison-adjusted funnel plot (**Figure 5C**) showed relatively even and symmetric distribution of study points across interventions. Although the regression line displayed slight slope, most studies remained within the funnel plot confidence bounds with no severe asymmetric distribution pattern, indicating absence of substantial systematic publication bias.

## 4 Discussion

To our knowledge, this is the first network meta-analysis to simultaneously evaluate the comparative efficacy of biologics across three difficult-to-treat sites, including scalp, nails, and palmoplantar areas, using complete or near-complete site-specific clearance as the primary outcome. Given the visibility, functional importance, and substantial quality-of-life impact of these sites, conventional PASI 75 or 90 endpoints often fail to capture meaningful patient benefit, prompting our adoption of a more stringent clinical benchmark (44). By focusing on stringent site-specific clearance endpoints, our study addresses an important evidence gap in the comparative effectiveness of IL-17 and IL-23 pathway inhibitors for refractory psoriasis phenotypes.

Overall, IL-17 inhibitors tended to rank highly for achieving rapid clearance at difficult-to-treat sites during the 12–16 week induction phase. Secukinumab and bimekizumab ranked highest for palmoplantar psoriasis, whereas bimekizumab and ixekizumab showed the most favorable rankings for nail psoriasis. However, the nail psoriasis findings should be interpreted cautiously because local inconsistency was detected for the ixekizumab-placebo comparison, and several comparisons were supported by low or very low certainty evidence. For scalp psoriasis, brodalumab achieved the highest SUCRA value, followed closely by ixekizumab and bimekizumab; however, the small ranking difference between brodalumab and ixekizumab, together with the single-study basis of the brodalumab estimate, warrants cautious interpretation. Notably, bimekizumab and ixekizumab consistently ranked in the top tier across all three domains, suggesting that they may be reasonable options when high-level clearance across several difficult-to-treat areas is a clinical priority.

The validity of indirect comparisons depends on the transitivity assumption. The included trials were broadly comparable with respect to adult psoriasis populations, site-specific disease involvement, and induction-phase assessment windows, with most outcomes assessed at 12–16 weeks. No major systematic imbalance was observed that would make treatment nodes clinically incomparable; however, potential effect modifiers were not perfectly balanced across treatment nodes, particularly prior biologic exposure, disease duration, baseline PASI, and racial composition; most trials enrolled predominantly White patients, whereas some included Asian cohorts. Meta-regression did not show consistent effect modification for most covariates, supporting the overall plausibility of transitivity. However, disease duration in the nail psoriasis network was associated with treatment effects in selected comparisons, indicating that residual intransitivity cannot be fully excluded. Accordingly, treatment rankings should be interpreted alongside certainty ratings, network geometry, inconsistency assessment, and sensitivity analyses rather than as fixed comparative hierarchies.

Compared with previous meta-analyses, our findings align directionally while providing updated site-specific evidence. A recent Asian-population meta-analysis of moderate-to-severe psoriasis reported that both IL-17 and IL-23 inhibitors showed favorable efficacy and safety, without establishing a clear class-level separation in overall PASI-based improvement (45). This broader evidence differs from our site-specific analysis in population scope, outcome definition, and clinical focus, as our study evaluated complete or near-complete clearance of scalp, nail, and palmoplantar lesions rather than overall skin improvement. For palmoplantar psoriasis, secukinumab ranked highest in our analysis, in line with prior systematic reviews and network meta-analyses (19, 20, 46). Differences from Spencer et al. (47), in which guselkumab ranked first, may partly reflect dose selection, as that analysis evaluated secukinumab 150 mg, whereas our analysis considered the standard 300 mg dose. For nail psoriasis, earlier meta-analyses suggested that ixekizumab had the greatest probability of achieving complete nail clearance, followed by brodalumab (48, 49). Our updated network, which included newer evidence for bimekizumab, suggests a shift in the ranking hierarchy, with bimekizumab ranked first, followed by ixekizumab and brodalumab. For scalp psoriasis, where sufficient dedicated head-to-head evidence remains lacking (50), brodalumab, ixekizumab, and bimekizumab also showed favorable SUCRA rankings in our analysis.

Mechanistically, our study suggests that for difficult-to-treat psoriatic lesions at palmoplantar, scalp, and nail sites, direct inhibition of the downstream effector IL-17 may confer clinical advantages over blocking the upstream regulator IL-23. IL-17A is the key effector molecule driving aberrant keratinocyte proliferation and inflammatory chemotaxis (11). IL-17 inhibitors directly interrupt this terminal inflammatory signal, rapidly curtailing the psoriatic inflammatory cascade and thereby excelling in speed of onset and depth of lesion clearance. Among these, bimekizumab simultaneously neutralizes both IL-17A and IL-17F. Because IL-17F exists at high concentrations in lesional skin and acts synergistically with IL-17A, this dual blockade may achieve deeper inflammatory suppression, effectively penetrating the thick stratum corneum of palmoplantar regions or the dense nail unit (51). Similarly, brodalumab blocks IL-17 receptor A (IL-17RA), effectively inhibiting signaling from IL-17A, F, C, and E isoforms, which likely underlies its exceptional performance in scalp psoriasis (52, 53). By contrast, IL-23 acts primarily upstream, maintaining Th17 cell survival and expansion. Blocking this target requires longer signal transmission time, resulting in relatively slower onset. The ECLIPSE study (54) demonstrated that guselkumab offers advantages over secukinumab in long-term efficacy maintenance and dosing convenience. Real-world data from Jung et al. (55) show that IL-17 inhibitors generally achieve higher early response rates than IL-23 inhibitors. Because our study relies predominantly on short-term RCT data from weeks 12–24, this mechanistic temporal difference likely explains why IL-17 inhibitors showed superior lesion clearance. Conversely, the IL PSO real-world study by Trovato et al. showed that IL-23p19 inhibitors, including risankizumab, guselkumab, and tildrakizumab 200 mg, maintained substantial 52-week effectiveness across high-impact sites, suggesting that long-term durability should also be considered when selecting biologics (56).

Beyond overall rankings, our analysis reveals clinical nuances relevant to managing patients with mixed phenotypes. In clinical practice, patients rarely present with isolated nail or scalp disease alone. For patients with concurrent severe scalp and nail involvement, our SUCRA rankings suggest that initiating treatment with bimekizumab or ixekizumab may offer the favorable probability of achieving clearance in both domains simultaneously, while acknowledging the uncertainty around several indirect comparisons. Conversely, although ustekinumab demonstrated satisfactory efficacy for palmoplantar psoriasis in our analysis, its performance was relatively modest for nail and scalp clearance, suggesting it may be less suitable for patients with extensive high-impact area involvement when rapid site-specific clearance is the principal goal.

Several limitations should be emphasized. First, the evidence networks were dominated by placebo-controlled trials, and direct head-to-head evidence between active biologics was limited. Consequently, many comparisons relied on indirect evidence, and treatment rankings should be interpreted alongside network geometry, inconsistency assessment, and evidence certainty. Second, the certainty of evidence varied substantially across comparisons. For nail psoriasis, local inconsistency was detected for the ixekizumab-placebo comparison, and 9 of 21 comparisons were rated as very low certainty (**Supplementary Table 15**). For scalp psoriasis, brodalumab relied on a single contributing study, and ixekizumab showed some variation in effect magnitude after exclusion of the UNCOVER_1_2_3 dataset. These findings support cautious interpretation of SUCRA-based rankings. Third, the primary endpoint required pragmatic harmonization of several site-specific instruments. Although these thresholds were clinically aligned and supported by prior site-specific NMAs, they are not strictly interchangeable. Residual heterogeneity in outcome definitions may therefore have influenced some effect estimates.

Fourth, most outcomes were assessed at 12–16 weeks. This induction-phase window may underestimate the longer-term efficacy of slower-acting agents, especially IL-23 inhibitors, and our findings should therefore be interpreted as short-term comparative efficacy rather than long-term disease control or maintenance efficacy. However, for nail psoriasis, the favorable IL-17 signal observed in our short-term analysis is supported by longer-term prospective real-world evidence, in which anti-IL-17A biologics showed greater mNAPSI improvement than other biologics through month 12, with ixekizumab demonstrating the greatest numerical improvement at month 12 (57). Fifth, the scope of this analysis was limited to scalp, nail, and palmoplantar psoriasis. Genital psoriasis, another clinically important high-impact site with substantial quality-of-life burden, was not evaluated. Therefore, our findings should not be generalized to all high-impact areas of psoriasis. Sixth, some difficult-to-treat site outcomes were derived from *post hoc* or subgroup-derived analyses rather than dedicated site-specific trials, which may introduce selective-reporting concerns. Finally, enrolled populations were predominantly White/Caucasian, limiting generalizability to other racial and ethnic groups, including Asian populations where phenotype distribution, background genetics, and treatment response may differ. Future head-to-head trials with prespecified scalp, nail, palmoplantar, and genital endpoints, longer follow-up, and more diverse populations are needed to confirm these comparative efficacy findings.

## 5 Conclusion

In summary, both IL-17 and IL-23 inhibitors were effective for difficult-to-treat psoriasis sites. Based on available induction-phase data, IL-17 inhibitors, particularly bimekizumab, ixekizumab, and brodalumab, tended to rank highest for complete or near-complete clearance of scalp, nail, and palmoplantar psoriasis. However, the certainty of evidence varied across comparisons, and several estimates were influenced by inconsistency or individual studies. Therefore, these rankings should be interpreted as probabilistic signals rather than definitive treatment hierarchies. Long-term, dedicated head-to-head trials with prespecified site-specific endpoints are needed to confirm these findings.

## Data Availability

The original contributions presented in the study are included in the article/**Supplementary Material**. Further inquiries can be directed to the corresponding author.
