# Supplementary material for: Short-term efficacy of biologics targeting the IL-17/IL-23 axis in scalp, nail, and palmoplantar psoriasis: a network meta-analysis of randomized controlled trials
Source: Front Immunol. 2026 Jul 22;17:1846290. doi: 10.3389/fimmu.2026.1846290 (PMC13437967; doi:10.3389/fimmu.2026.1846290)
Supplement: Supplementary file 1 [file Table1.docx]

**Short-Term Efficacy of Biologics Targeting the IL-17/IL-23 Axis in Scalp, Nail, and Palmoplantar Psoriasis: A Network Meta-Analysis of Randomized Controlled Trials**

| **Table of Contents** | | |
| --- | --- | --- |
| **Title** | Content | page |
| **Table S1** | PRISMA NMA Checklist of Items to Include When Reporting a Systematic Review Involving a Network Meta-analysis | 2-6 |
| **Table S2** | Literature Search Strategy | 6-8 |
| **Table S3** | Outcome reporting status of site-specific endpoints in included trials | 9-10 |
| **Table S4** | Node-Splitting Analysis of Inconsistency for Palmoplantar Psoriasis | 11 |
| **Table S5** | SUCRA Values for Treatments in Psoriasis | 11 |
| **Table S6** | Ranking Profiles in the Bayesian Network Meta-analysis (First-column) | 12 |
| Table S7 | GRADE Certainty of Evidence Profile for Palmoplantar Psoriasis Outcomes | 13-14 |
| **Table S8** | Sensitivity Analysis Results for Complete Clearance in Palmoplantar Psoriasis | 15-16 |
| Table S9 | Meta-Regression Analysis of Age for Complete Clearance in Palmoplantar Psoriasis | 17 |
| **Table S10** | Meta-Regression Analysis of Previous Bio Therapy for Complete Clearance in Palmoplantar Psoriasis | 18 |
| Table S11 | Meta-Regression Analysis of Disease Duration for Complete Clearance in Palmoplantar Psoriasis | 19 |
| **Table S12** | Meta-Regression Analysis of Baseline PASI for Complete Clearance in Palmoplantar Psoriasis | 20 |
| Table S13 | Meta-Regression Analysis of Timepoint for Complete Clearance in Palmoplantar Psoriasis | 21 |
| **Table S14** | Node-Splitting Analysis of Inconsistency for Nail Psoriasis | 22 |
| Table S15 | GRADE Certainty of Evidence Profile for Nail Psoriasis Outcomes | 23-25 |
| **Table S16** | Sensitivity Analysis Results for Complete Clearance in Nail Psoriasis | 26-27 |
| Table S17 | Meta-Regression Analysis of Age for Complete Clearance in Nail Psoriasis | 28 |
| **Table S18** | Meta-Regression Analysis of Previous Bio Therapy for Complete Clearance in Nail Psoriasis | 29 |
| Table S19 | Meta-Regression Analysis of Disease Duration for Complete Clearance in Nail Psoriasis | 30 |
| **Table S20** | Meta-Regression Analysis of Baseline PASI for Complete Clearance in Nail Psoriasis | 31 |
| Table S21 | Meta-Regression Analysis of Timepoint for Complete Clearance in Nail Psoriasis | 32 |
| **Table S22** | GRADE Certainty of Evidence Profile for Scalp Psoriasis Outcomes | 33-35 |
| Table S23 | Sensitivity Analysis Results for Complete Clearance in Scalp Psoriasis | 36-38 |
| **Table S24** | Meta-Regression Analysis of Age for Complete Clearance in Scalp Psoriasis | 39 |
| Table S25 | Meta-Regression Analysis of Previous Bio Therapy for Complete Clearance in Scalp Psoriasis | 40-41 |
| **Table S26** | Meta-Regression Analysis of Disease Duration for Complete Clearance in Scalp Psoriasis | 41-42 |
| Table S27 | Meta-Regression Analysis of Baseline PASI for Complete Clearance in Scalp Psoriasis | 42-43 |
| **Table S28** | Meta-Regression Analysis of Timepoint for Complete Clearance in Scalp Psoriasis | 43-44 |
| Figure S1 | Risk of Bias Summary for Included Randomized Controlled Trials Using Cochrane RoB 2.0 Tool | 45 |
| **Figure S2** | Inconsistency Plots Assessing Loop-Specific Heterogeneity for Palmoplantar Psoriasis Network | 46 |
| Figure S3 | Inconsistency Plots Assessing Loop-Specific Heterogeneity for Nail Psoriasis Network | 46 |

***Supplementary Tables***

***Supplement 1 Checklist & Search Strategy***

**Table S1 PRISMA NMA Checklist of Items to Include When Reporting a Systematic Review Involving a Network Meta-analysis**

| Section/Topic | Item # | Checklist Item | Reported location |
| --- | --- | --- | --- |
| TITLE |  |  |  |
| Title | 1 | Identify the report as a systematic review *incorporating*  anetwork meta-analysis (or related form of meta-analysis). | Title |
|  |  |  |  |
| ABSTRACT |  |  |  |
| Structured summary | 2 | Provide a structured summary including, as applicable:  **Background:** main objectives  **Methods:** data sources; study eligibility criteria, participants, and interventions; study appraisal; and *synthesis methods, such as network meta-analysis.*  **Results:** number of studies and participants identified; summary estimates with corresponding confidence/credible intervals; treatment rankings may also be discussed. Authors may choose to summarize pairwise comparisons against a chosen treatment included in their analyses for brevity.  **Discussion/Conclusions:** limitations; conclusions and implications of findings.  **Other:** systematic review registration number with registry name. | Abstract |
|  |  |  |  |
| INTRODUCTION |  |  |  |
| Rationale | 3 | Describe the rationale for the review in the context of what is already known*, including mention of why a network meta-analysis has been conducted.* | Introduction |
| Objectives | 4 | Provide an explicit statement of questions being addressed, with reference to participants, interventions, comparisons, outcomes, and study design (PICOS). | Introduction |
|  |  |  |  |
| METHODS |  |  |  |
| Protocol and registration | 5 | Indicate whether a review protocol exists and if and where it can be accessed (e.g., Web address); and, if available, provide registration information, including registration number. | Methods; PROSPERO CRD420251271255 |
| Eligibility criteria | 6 | Specify study characteristics (e.g., PICOS, length of follow-up) and report characteristics (e.g., years considered, language, publication status) used as criteria for eligibility, giving rationale. *Clearly describe eligible treatments included in the treatment network, and note whether any have been clustered or merged into the same node (with justification).* | Section 2.2 |
| Information sources | 7 | Describe all information sources (e.g., databases with dates of coverage, contact with study authors to identify additional studies) in the search and date last searched. | Section 2.1 |
| Search | 8 | Present full electronic search strategy for at least one database, including any limits used, such that it could be repeated. | Section 2.1;  Table S2 |
| Study selection | 9 | State the process for selecting studies (i.e., screening, eligibility, included in systematic review, and, if applicable, included in the meta-analysis). | Section 2.2;  Figure 1 |
| Data collection process | 10 | Describe method of data extraction from reports (e.g., piloted forms, independently, in duplicate) and any processes for obtaining and confirming data from investigators. | Section 2.3 |
| Data items | 11 | List and define all variables for which data were sought (e.g., PICOS, funding sources) and any assumptions and simplifications made. | Section 2.3;  Tables 1–2 |
| Geometry of the network | S1 | Describe methods used to explore the geometry of the treatment network under study and potential biases related to it. This should include how the evidence base has been graphically summarized for presentation, and what characteristics were compiled and used to describe the evidence base to readers. | Section 2.4;  Figure 2 |
| Risk of bias within individual studies | 12 | Describe methods used for assessing risk of bias of individual studies (including specification of whether this was done at the study or outcome level), and how this information is to be used in any data synthesis. | Section 2.3;  Figure S1;  Table S3 |
| Summary measures | 13 | State the principal summary measures (e.g., risk ratio, difference in means). Also describe the use of additional summary measures assessed, such as treatment rankings and surface under the cumulative ranking curve (SUCRA) values, as well as modified approaches used to present summary findings from meta-analyses. | Section 2.4 |
| Planned methods of analysis | 14 | Describe the methods of handling data and combining results of studies for each network meta-analysis. This should include, but not be limited to:   - Handling of multi-arm trials; - Selection of variance structure; - Selection of prior distributions in Bayesian analyses; and - Assessment of model fit. | Section 2.4 |
| Assessment of Inconsistency | S2 | Describe the statistical methods used to evaluate the agreement of direct and indirect evidence in the treatment network(s) studied. Describe efforts taken to address its presence when found. | Section 2.4; Tables S4 and S14; Figures S2–S3 |
| Risk of bias across studies | 15 | Specify any assessment of risk of bias that may affect the cumulative evidence (e.g., publication bias, selective reporting within studies). | Sections 2.3–2.4; Table S3; Figure 5 |
| Additional analyses | 16 | Describe methods of additional analyses if done, indicating which were pre-specified. This may include, but not be limited to, the following:   - Sensitivity or subgroup analyses; - Meta-regression analyses; - Alternative formulations of the treatment network; and - Use of alternative prior distributions for Bayesian analyses (if applicable). | Section 2.4 |
| RESULTS† |  |  |  |
| Study selection | 17 | Give numbers of studies screened, assessed for eligibility, and included in the review, with reasons for exclusions at each stage, ideally with a flow diagram. | Section 3.1; Figure 1 |
| Presentation of network structure | S3 | Provide a network graph of the included studies to enable visualization of the geometry of the treatment network. | Sections 3.2–3.4;  Figure 2 |
| Summary of network geometry | S4 | Provide a brief overview of characteristics of the treatment network. This may include commentary on the abundance of trials and randomized patients for the different interventions and pairwise comparisons in the network, gaps of evidence in the treatment network, and potential biases reflected by the network structure. | Sections 3.2–3.4 |
| Study characteristics | 18 | For each study, present characteristics for which data were extracted (e.g., study size, PICOS, follow-up period) and provide the citations. | Section 3.1;  Tables 1–2 |
| Risk of bias within studies | 19 | Present data on risk of bias of each study and, if available, any outcome level assessment. | Section 3.1; Figure S1; Table S3 |
| Results of individual studies | 20 | For all outcomes considered (benefits or harms), present, for each study: 1) simple summary data for each intervention group, and 2) effect estimates and confidence intervals. *Modified approaches may be needed to deal with information from larger networks.* | Sections 3.2–3.4; Figure 3; Table 3 |
| Synthesis of results | 21 | Present results of each meta-analysis done, including confidence/credible intervals. In larger networks, authors may focus on comparisons versus a particular comparator (e.g. placebo or standard care), with full findings presented in an appendix. League tables and forest plots may be considered to summarize pairwise comparisons. If additional summary measures were explored (such as treatment rankings), these should also be presented. | Sections 3.2–3.4;  Figure 3; Figure 4; Table 3; Tables S5–S6 |
| Exploration for inconsistency | S5 | Describe results from investigations of inconsistency. This may include such information as measures of model fit to compare consistency and inconsistency models, *P* values from statistical tests, or summary of inconsistency estimates from different parts of the treatment network. | Sections 3.2 and 3.3; Tables S4 and S14; Figures S2–S3 |
| Risk of bias across studies | 22 | Present results of any assessment of risk of bias across studies for the evidence base being studied. | Sections 3.2–3.4;  Figure 5 |
| Results of additional analyses | 23 | Give results of additional analyses, if done (e.g., sensitivity or subgroup analyses, meta-regression analyses*, alternative network geometries studied, alternative choice of prior distributions for Bayesian analyses,* and so forth). | Sections 3.2–3.4; Tables S8–S13, S16–S21, and S23–S28 |
|  |  |  |  |
| DISCUSSION |  |  |  |
| Summary of evidence | 24 | Summarize the main findings, including the strength of evidence for each main outcome; consider their relevance to key groups (e.g., healthcare providers, users, and policy-makers). | Discussion; Table 3; Tables S7, S15, and S22 |
| Limitations | 25 | Discuss limitations at study and outcome level (e.g., risk of bias), and at review level (e.g., incomplete retrieval of identified research, reporting bias). *Comment on the validity of the assumptions, such as transitivity and consistency. Comment on any concerns regarding network geometry (e.g., avoidance of certain comparisons).* | Discussion |
| Conclusions | 26 | Provide a general interpretation of the results in the context of other evidence, and implications for future research. | Conclusion |
|  |  |  |  |
| FUNDING |  |  |  |
| Funding | 27 | Describe sources of funding for the systematic review and other support (e.g., supply of data); role of funders for the systematic review. This should also include information regarding whether funding has been received from manufacturers of treatments in the network and/or whether some of the authors are content experts with professional conflicts of interest that could affect use of treatments in the network. | Funding statement |

PICOS = population, intervention, comparators, outcomes, study design.

* Text in italics indicate S wording specific to reporting of network meta-analyses that has been added to guidance from the PRISMA statement.

† Authors may wish to plan for use of appendices to present all relevant information in full detail for items in this section.

| **Table S2. Literature Search Strategy** | |
| --- | --- |
| Pubmed | (("Psoriasis"[Mesh] OR psoriasis[tiab] OR psoria*[tiab])  AND ("Interleukin-23/antagonists and inhibitors"[Mesh] OR "Interleukin 23 subunit p19"[Mesh] OR "Interleukin-17/antagonists and inhibitors"[Mesh] OR "IL 23 inhibitor*"[tiab] OR "IL 17 inhibitor*"[tiab] OR "Interleukin 23 inhibitor*"[tiab] OR "Interleukin 17 inhibitor*"[tiab] OR "IL 23 blocker*"[tiab] OR "IL 17 blocker*"[tiab] OR "Interleukin 23 blocker*"[tiab] OR "Interleukin 17 blocker*"[tiab] OR "IL 23 antagonist*"[tiab] OR "IL 17 antagonist*"[tiab] OR "Interleukin 23 antagonist*"[tiab] OR "Interleukin 17 antagonist*"[tiab] OR "Anti IL 23*"[tiab] OR "Anti IL 17*"[tiab] OR secukinumab[tiab] OR cosentyx[tiab] OR ixekizumab[tiab] OR taltz[tiab] OR brodalumab[tiab] OR siliq[tiab] OR ustekinumab[tiab] OR stelara[tiab] OR guselkumab[tiab] OR tremfya[tiab] OR tildrakizumab[tiab] OR ilumya[tiab] OR risankizumab[tiab] OR skyrizi[tiab] OR mirikizumab[tiab] OR bimekizumab[tiab]))  AND (palmoplantar[tiab] OR palm*[tiab] OR palmar[tiab] OR plantar*[tiab] OR hand[tiab] OR hands[tiab] OR foot[tiab] OR feet[tiab] OR nail[tiab] OR "Nail Diseases"[Mesh] OR onych*[tiab] OR scalp[tiab] OR hairline[tiab])  Filters: Randomized Controlled Trial |
|  |  |
|  |  |
|  |  |
|  |  |
|  |  |
|  |  |
| Web of Science | TS=(psoriasis OR psoria*) AND TS=(palmoplantar OR palm* OR palmar OR plantar* OR hand OR hands* OR foot* OR feet* OR nail* OR onycho* OR scalp* OR hairline* OR "difficult-to-treat areas") AND TS=("IL 23 inhibitor*" OR "IL 17 inhibitor*" OR "interleukin 23 inhibitor*" OR "interleukin 17 inhibitor*" OR "IL 23 blocker*" OR "IL 17 blocker*" OR "interleukin 23 blocker*" OR "interleukin 17 blocker*" OR "IL 23 antagonist*" OR "IL 17 antagonist*" OR "interleukin 23 antagonist*" OR "interleukin 17 antagonist*" OR "anti IL 23*" OR "anti IL 17*" OR secukinumab OR cosentyx OR ixekizumab OR taltz OR brodalumab OR siliq OR ustekinumab OR stelara OR guselkumab OR tremfya OR tildrakizumab OR ilumya OR risankizumab OR skyrizi OR mirikizumab OR bimekizumab) AND  TS=(random* OR trial OR "double blind" OR placebo) |
|  |  |
|  |  |
|  |  |
|  |  |
|  |  |
|  |  |
|  |  |
| Cochrane | ([mh "Psoriasis"] OR psoriasis:ti,ab,kw OR psoria*:ti,ab,kw) AND (palmoplantar:ti,ab,kw OR palm*:ti,ab,kw OR palmar:ti,ab,kw OR plantar*:ti,ab,kw OR hand:ti,ab,kw OR hands:ti,ab,kw OR foot:ti,ab,kw OR feet:ti,ab,kw OR nail:ti,ab,kw OR onych*:ti,ab,kw OR scalp:ti,ab,kw OR hairline:ti,ab,kw OR "difficult-to-treat areas":ti,ab,kw) AND ([mh "Interleukin-23/antagonists and inhibitors"] OR [mh "Interleukin 23 subunit p19"] OR [mh "Interleukin-17/antagonists and inhibitors"] OR "IL 23 inhibitor*":ti,ab,kw OR "IL 17 inhibitor*":ti,ab,kw OR "interleukin 23 inhibitor*":ti,ab,kw OR "interleukin 17 inhibitor*":ti,ab,kw OR "IL 23 blocker*":ti,ab,kw OR "IL 17 blocker*":ti,ab,kw OR "interleukin 23 blocker*":ti,ab,kw OR "interleukin 17 blocker*":ti,ab,kw OR "IL 23 antagonist*":ti,ab,kw OR "IL 17 antagonist*":ti,ab,kw OR "interleukin 23 antagonist*":ti,ab,kw OR "interleukin 17 antagonist*":ti,ab,kw OR "anti IL 23*":ti,ab,kw OR "anti IL 17*":ti,ab,kw OR secukinumab:ti,ab,kw OR cosentyx:ti,ab,kw OR ixekizumab:ti,ab,kw OR taltz:ti,ab,kw OR brodalumab:ti,ab,kw OR siliq:ti,ab,kw OR ustekinumab:ti,ab,kw OR stelara:ti,ab,kw OR guselkumab:ti,ab,kw OR tremfya:ti,ab,kw OR tildrakizumab:ti,ab,kw OR ilumya:ti,ab,kw  OR risankizumab:ti,ab,kw OR skyrizi:ti,ab,kw OR mirikizumab:ti,ab,kw OR bimekizumab:ti,ab,kw) |
|  |  |
|  |  |
|  |  |
| Embase | (psoriasis/exp OR psoriasis:ti,ab OR psoria*:ti,ab)  AND  (palmoplantar:ti,ab OR palm*:ti,ab OR palmar:ti,ab OR plantar*:ti,ab OR hand:ti,ab OR hands:ti,ab OR foot:ti,ab OR feet:ti,ab OR nail:ti,ab OR onycho*:ti,ab OR scalp:ti,ab OR hairline:ti,ab OR "difficult-to-treat areas")  AND  ('interleukin 23 inhibitor'/exp OR 'interleukin 23 subunit p19'/exp OR 'interleukin 17 inhibitor'/exp OR 'IL 23 inhibitor*':ti,ab OR 'IL 17 inhibitor*':ti,ab OR 'interleukin 23 inhibitor*':ti,ab OR 'interleukin 17 inhibitor*':ti,ab OR 'IL 23 blocker*':ti,ab OR 'IL 17 blocker*':ti,ab OR 'interleukin 23 blocker*':ti,ab OR 'interleukin 17 blocker*':ti,ab OR 'IL 23 antagonist*':ti,ab OR 'IL 17 antagonist*':ti,ab OR 'interleukin 23 antagonist*':ti,ab OR 'interleukin 17 antagonist*':ti,ab OR 'anti IL 23*':ti,ab OR 'anti IL 17*':ti,ab OR secukinumab:ti,ab OR osentyx:ti,ab  OR ixekizumab:ti,ab OR taltz:ti,ab OR brodalumab:ti,ab OR siliq:ti,ab  OR ustekinumab:ti,ab OR stelara:ti,ab OR guselkumab:ti,ab OR tremfya:ti,ab OR tildrakizumab:ti,ab OR ilumya:ti,ab OR risankizumab:ti,ab OR skyrizi:ti,ab OR mirikizumab:ti,ab OR bimekizumab:ti,ab)  AND  1.'controlled clinical trial'/exp  2. (random* OR placebo*):ti,ab  3. trial:ti  4. (#1 or #2 OR #3) AND [embase]/lim |
|  |  |
|  |  |
|  |  |
|  |  |
|  |  |
|  |  |
|  |  |
|  |  |
|  |  |
|  |  |

| **Table S3 Outcome reporting status of site-specific endpoints in included trials** | | | | |
| --- | --- | --- | --- | --- |
| **Study / dataset** | **Registration No.** | **Target area** | **Outcome used in NMA** | **Outcome reporting status** |
| Menter et al., 2017 / UNCOVER-1, -2, -3 | NCT01474512, NCT01597245, NCT01646177 | Palmoplantar | PPASI100 | Post hoc subgroup analysis |
| Papp et al., 2018 / UNCOVER-1, -2, -3 | NCT01474512, NCT01597245, NCT01646177 | Nail | NAPSI=0 | Post hoc subgroup analysis |
| Reich et al., 2016 / UNCOVER-1, -2, -3 | NCT01474512, NCT01597245, NCT01646177 | Scalp | PSSI100 | Post hoc subgroup analysis |
| Gottlieb et al., 2017 / GESTURE | NCT01806597 | Palmoplantar | ppIGA0/1 | Prespecified primary endpoint |
| Lebwohl et al., 2024 / IMMprint | NCT04713592 | Palmoplantar | ppIGA0/1 | Prespecified primary site-specific endpoint |
| Passeron et al., 2023 / G-PLUS | NCT03998683 | Palmoplantar | ppIGA0/1 | Prespecified site-specific endpoint, not primary |
| Gordon et al., 2021 / BE READY | NCT03410992 | Palmoplantar, nail, scalp | pp-IGA0/1, mNAPSI100, ss-IGA/Scalp IGA0/1 | Protocol-defined site-specific endpoint, not co-primary |
| Reich et al., 2021 / BE VIVID | NCT03370133 | Palmoplantar, nail, scalp | pp-IGA0/1, mNAPSI100, ss-IGA/Scalp IGA0/1 | Protocol-defined site-specific endpoint, not co-primary |
| Reich et al., 2016 / VOYAGE 2 | NCT02207244 | Palmoplantar, nail, scalp | hf-PGA0/1, f-PGA0/1, ss-IGA0/1 | Post hoc regional analysis; outcomes collected prospectively |
| Blauvelt et al., 2017 / VOYAGE 1 | NCT02207231 | Palmoplantar, nail, scalp | hf-PGA0/1, f-PGA0/1, ss-IGA0/1 | Post hoc regional analysis; outcomes collected prospectively |
| Li et al., 2024 / Chinese ixekizumab trial | NCT03364309 | Palmoplantar, scalp | PPASI100, PSSI100 | Post hoc subgroup analysis |
| Elewski et al., 2020 / AMAGINE-1 | NCT01708590 | Scalp | PSSI100 | Post hoc subgroup analysis |
| Elewski et al., 2020 / AMAGINE-2/-3 | NCT01708603, NCT01708629 | Nail | NAPSI=0 | Post hoc subgroup analysis |
| Mease et al., 2017 / SPIRIT-P1 | NCT01695239 | Nail | NAPSI=0 | Protocol-defined nail endpoint, not primary |
| Wasel et al., 2020 / IXORA-S | NCT02561806 | Nail | NAPSI=0 | Post hoc nail subgroup analysis |
| Coates et al., 2025 / KEEPsAKE 1/2 | NCT03675308 | Nail | f-PGA0/1 | Post hoc subgroup analysis |
| Blauvelt et al., 2021 / IXORA-R | NCT03573323 | Nail | f-PGA0/1 | Protocol-defined nail endpoint, not primary |
| Bagel et al., 2017 | NCT02267135 | Scalp | ss-IGA0/1 | Prespecified scalp endpoint |
| Stein Gold et al., 2025 / SPECTREM | NCT06039189 | Scalp | ss-IGA0/1 | Prespecified high-impact-site scalp endpoint |
| Song et al., 2025 / UnlIMMited | NCT05969223 | Scalp | ss-IGA0/1 | Prespecified site-specific endpoint |
| Ohtsuki et al., 2018 | NCT02325219 | Scalp | ss-IGA0/1 | Protocol-defined scalp endpoint, not primary |
| McMichael et al., 2025 / VISIBLE Cohort B | NCT05272150 | Scalp | ss-IGA0/1 | Prespecified co-primary site-specific endpoint |
| Gebauer et al., 2024 | NCT03897088 | Scalp | ss-IGA0/1 | Prespecified scalp endpoint |

***Supplement 2	Palmoplantar Psoriasis***

| **Table S4.** Node-Splitting Analysis of Inconsistency for Palmoplantar Psoriasis | | | | | |
| --- | --- | --- | --- | --- | --- |
| Comparison (Side) | Direct Evidence Coef. (SE) | Indirect Evidence Coef. (SE) | | Inconsistency Factor (Difference) Coef. (SE) | *P*-value |
| Bimekizumab vs. Placebo | -3.00 (0.61) | | -1.61 (259.13) | -1.39 (259.13) | 0.996 |
| Bimekizumab vs. Ustekinumab | 0.14 (0.88) | | -1.60 (2.49) | 1.74 (2.61) | 0.505 |
| Guselkumab vs. Placebo | -2.33 (0.48) | | -5.12 (759.22) | 2.79 (759.22) | 0.997 |
| Ixekizumab vs. Placebo | -2.66 (0.60) | | -5.22 (938.92) | 2.56 (938.92) | 0.998 |
| Risankizumab vs. Placebo | 0.96 (0.77) | | 5.87 (1448.60) | -4.91 (1448.60) | 0.997 |
| Secukinumab vs. Placebo | 3.51 (1.24) | | 4.21 (1651.48) | -0.70 (1651.48) | 1 |
| Ustekinumab vs. Placebo | 2.73 (0.95) | | 4.47 (2.41) | -1.74 (2.61) | 0.505 |

SE: Standard Error.

| **Table S5.** SUCRA Values for Treatments in Psoriasis | | | |
| --- | --- | --- | --- |
| Treatment | SUCRA | PrBest | MeanRank |
| **Palmoplantar Psoriasis** | | | |
| Secukinumab | 79.7 | 52.5 | 2.2 |
| Bimekizumab | 72.2 | 16.4 | 2.7 |
| Ustekinumab | 69.7 | 19.7 | 2.8 |
| Ixekizumab | 59.7 | 9.2 | 3.4 |
| Guselkumab | 48.3 | 2.1 | 4.1 |
| Risankizumab | 18.5 | 0.1 | 5.9 |
| Placebo | 1.8 | 0 | 6.9 |
| **Nail Psoriasis** | | | |
| Bimekizumab | 78.9 | 36 | 2.3 |
| Ixekizumab | 77.2 | 22.9 | 2.4 |
| Brodalumab | 67.5 | 28 | 2.9 |
| Guselkumab | 57 | 5.2 | 3.6 |
| Risankizumab | 47.9 | 7.8 | 4.1 |
| Ustekinumab | 18.3 | 0 | 5.9 |
| Placebo | 3.3 | 0 | 6.8 |
| **Scalp Psoriasis** | | | |
| Brodalumab | 87.7 | 59.1 | 2 |
| Ixekizumab | 86.9 | 25.2 | 2 |
| Bimekizumab | 69 | 7 | 3.5 |
| Guselkumab | 65.1 | 0.6 | 3.8 |
| Secukinumab | 52.9 | 7.7 | 4.8 |
| Ustekinumab | 32 | 0 | 6.4 |
| Tildrakizumab | 30.9 | 0.2 | 6.5 |
| Risankizumab | 25.5 | 0.1 | 7 |
| Placebo | 0 | 0 | 9 |

| Table S6. Ranking Profiles in the Bayesian Network Meta-analysis（First-column） | | | | | | | | | |
| --- | --- | --- | --- | --- | --- | --- | --- | --- | --- |
| Treatment | Best | 2nd | 3rd | 4th | 5th | 6th | 7th | 8th | 9th (Worst) |
| **Palmoplantar Psoriasis** |  |  |  |  |  |  |  |  |  |
| Secukinumab | 52.5 | 13.4 | 11.9 | 8.5 | 10.1 | 3.5 | 0.2 | — | — |
| Ustekinumab | 19.7 | 27.9 | 20.8 | 16.4 | 12.9 | 2.2 | 0 | — | — |
| Bimekizumab | 16.4 | 31.6 | 29 | 15.5 | 6.7 | 0.8 | 0 | — | — |
| Ixekizumab | 9.2 | 18.4 | 21.1 | 27.5 | 21 | 2.9 | 0 | — | — |
| Guselkumab | 2.1 | 8.1 | 16.1 | 29.6 | 39.4 | 4.7 | 0 | — | — |
| Risankizumab | 0.1 | 0.6 | 1.2 | 2.5 | 9.9 | 75.3 | 10.4 | — | — |
| Placebo | 0 | 0 | 0 | 0 | 0 | 10.6 | 89.4 | — | — |
| **Nail Psoriasis** |  |  |  |  |  |  |  |  |  |
| Bimekizumab | 36 | 26.6 | 18.2 | 13.1 | 5.8 | 0.2 | 0.1 | — | — |
| Brodalumab | 28 | 17.2 | 14.6 | 16.7 | 19.9 | 2.8 | 0.8 | — | — |
| Ixekizumab | 22.9 | 33.8 | 29.5 | 10.9 | 2.8 | 0.1 | 0 | — | — |
| Risankizumab | 7.8 | 9 | 12.6 | 22 | 31.7 | 15.5 | 1.3 | — | — |
| Guselkumab | 5.2 | 13.3 | 24.7 | 34.1 | 20 | 2.6 | 0 | — | — |
| Ustekinumab | 0 | 0 | 0.3 | 3.3 | 18.2 | 62.2 | 16.1 | — | — |
| Placebo | 0 | 0 | 0 | 0.1 | 1.5 | 16.6 | 81.8 | — | — |
| **Scalp Psoriasis** |  |  |  |  |  |  |  |  |  |
| Brodalumab | 59.1 | 14.8 | 9.5 | 7.7 | 5 | 2.3 | 1.3 | 0.3 | 0 |
| Ixekizumab | 25.2 | 50.3 | 19.8 | 4.1 | 0.6 | 0 | 0 | 0 | 0 |
| Bimekizumab | 7 | 17.4 | 25.9 | 26.2 | 17.8 | 5 | 0.7 | 0 | 0 |
| Secukinumab | 7.7 | 9.8 | 10.6 | 12.4 | 20.6 | 16.8 | 12.3 | 9.7 | 0 |
| Guselkumab | 0.6 | 6.7 | 30.9 | 40 | 18.4 | 3.2 | 0.3 | 0 | 0 |
| Tildrakizumab | 0.2 | 0.7 | 2.1 | 4.3 | 13 | 23.8 | 27.5 | 28.4 | 0 |
| Risankizumab | 0.1 | 0.3 | 0.8 | 2.5 | 8.7 | 17 | 27.1 | 43.5 | 0 |
| Ustekinumab | 0 | 0 | 0.4 | 2.7 | 15.9 | 32 | 30.8 | 18.1 | 0 |
| Placebo | 0 | 0 | 0 | 0 | 0 | 0 | 0 | 0 | 100 |

Table S7. GRADE Certainty of Evidence Profile for Palmoplantar Psoriasis Outcomes

| **Comparison** | **Number of studies** | **Within-study bias** | **Reporting bias** | **Indirectness** | **Imprecision** | **Heterogeneity** | **Incoherence** | **Confidence rating** | **Reason(s) for downgrading** |
| --- | --- | --- | --- | --- | --- | --- | --- | --- | --- |
| Bimekizumab:Placebo | 2 | No concerns | Low risk | No concerns | No concerns | No concerns | No concerns | High | [] |
| Bimekizumab:Ustekinumab | 1 | No concerns | Low risk | No concerns | Some concerns | No concerns | No concerns | Moderate | ["Imprecision"] |
| Guselkumab:Placebo | 3 | No concerns | Low risk | No concerns | No concerns | Some concerns | No concerns | Moderate | ["Heterogeneity"] |
| Ixekizumab:Placebo | 2 | Some concerns | Low risk | No concerns | No concerns | Some concerns | No concerns | Low | ["Within-study bias","Heterogeneity"] |
| Placebo:Risankizumab | 1 | No concerns | Low risk | No concerns | Some concerns | No concerns | No concerns | Moderate | ["Imprecision"] |
| Placebo:Secukinumab | 1 | No concerns | Low risk | No concerns | No concerns | Some concerns | No concerns | Moderate | ["Heterogeneity"] |
| Placebo:Ustekinumab | 1 | No concerns | Low risk | No concerns | No concerns | Some concerns | No concerns | Moderate | ["Heterogeneity"] |
| Bimekizumab:Guselkumab | 0 | No concerns | Low risk | No concerns | Some concerns | No concerns | No concerns | Moderate | ["Imprecision"] |
| Bimekizumab:Ixekizumab | 0 | No concerns | Low risk | No concerns | Some concerns | No concerns | No concerns | Moderate | ["Imprecision"] |
| Bimekizumab:Risankizumab | 0 | No concerns | Low risk | No concerns | No concerns | Some concerns | No concerns | Moderate | ["Heterogeneity"] |
| Bimekizumab:Secukinumab | 0 | No concerns | Low risk | No concerns | Some concerns | No concerns | No concerns | Moderate | ["Imprecision"] |
| Guselkumab:Ixekizumab | 0 | No concerns | Low risk | No concerns | Some concerns | No concerns | No concerns | Moderate | ["Imprecision"] |
| Guselkumab:Risankizumab | 0 | No concerns | Low risk | No concerns | Some concerns | No concerns | No concerns | Moderate | ["Imprecision"] |
| Guselkumab:Secukinumab | 0 | No concerns | Low risk | No concerns | Some concerns | No concerns | No concerns | Moderate | ["Imprecision"] |
| Guselkumab:Ustekinumab | 0 | No concerns | Low risk | No concerns | Some concerns | No concerns | No concerns | Moderate | ["Imprecision"] |
| Ixekizumab:Risankizumab | 0 | No concerns | Low risk | No concerns | Some concerns | No concerns | No concerns | Moderate | ["Imprecision"] |
| Ixekizumab:Secukinumab | 0 | No concerns | Low risk | No concerns | Some concerns | No concerns | No concerns | Moderate | ["Imprecision"] |
| Ixekizumab:Ustekinumab | 0 | No concerns | Low risk | No concerns | Some concerns | No concerns | No concerns | Moderate | ["Imprecision"] |
| Risankizumab:Secukinumab | 0 | No concerns | Low risk | No concerns | Some concerns | No concerns | No concerns | Moderate | ["Imprecision"] |
| Risankizumab:Ustekinumab | 0 | No concerns | Low risk | No concerns | Some concerns | Some concerns | No concerns | Low | ["Imprecision",  "Heterogeneity"] |
| Secukinumab:Ustekinumab | 0 | No concerns | Low risk | No concerns | Some concerns | No concerns | No concerns | Moderate | ["Imprecision"] |

| **Table S8. Sensitivity analysis results for complete clearance in palmoplantar psoriasis** | | | | | |
| --- | --- | --- | --- | --- | --- |
| **dropped_id** | **comparison** | **effect size** | **lower confidence interval** | **upper confidence interval** | **Connected Network Status** |
| NCT01806597 | Bimekizumab VS Placebo | 20.178319 | 6.108919 | 66.65084 | 1 |
| NCT01806597 | Guselkumab VS Placebo | 10.284711 | 4.032978 | 26.22759 | 1 |
| NCT01806597 | Ixekizumab VS Placebo | 14.28766 | 4.389424 | 46.50661 | 1 |
| NCT01806597 | Risankizumab VS Placebo | 2.6071429 | 0.575652 | 11.80781 | 1 |
| NCT01806597 | Secukinumab VS Placebo | 19.539642 | 3.882907 | 98.32778 | 1 |
| NCT02207231 | Bimekizumab VS Placebo | 20.242267 | 5.560867 | 73.68444 | 1 |
| NCT02207231 | Guselkumab VS Placebo | 8.0441001 | 2.328346 | 27.7912 | 1 |
| NCT02207231 | Ixekizumab VS Placebo | 14.337714 | 3.988847 | 51.53621 | 1 |
| NCT02207231 | Risankizumab VS Placebo | 2.6071429 | 0.494484 | 13.74603 | 1 |
| NCT02207231 | Secukinumab VS Placebo | 33.5 | 2.675584 | 419.4412 | 1 |
| NCT02207231 | Ustekinumab VS Placebo | 19.466648 | 3.410835 | 111.1019 | 1 |
| NCT02207244 | Bimekizumab VS Placebo | 20.157656 | 6.269101 | 64.81489 | 1 |
| NCT02207244 | Guselkumab VS Placebo | 7.0412198 | 2.253219 | 22.00353 | 1 |
| NCT02207244 | Ixekizumab VS Placebo | 14.271449 | 4.506866 | 45.19199 | 1 |
| NCT02207244 | Risankizumab VS Placebo | 2.6071429 | 0.600867 | 11.31231 | 1 |
| NCT02207244 | Secukinumab VS Placebo | 33.5 | 3.02787 | 370.64 | 1 |
| NCT02207244 | Ustekinumab VS Placebo | 19.563337 | 4.025412 | 95.07701 | 1 |
| NCT03364309 | Bimekizumab VS Placebo | 20.271111 | 5.286046 | 77.73635 | 1 |
| NCT03364309 | Guselkumab VS Placebo | 10.221024 | 3.532505 | 29.57372 | 1 |
| NCT03364309 | Ixekizumab VS Placebo | 11.953247 | 1.97279 | 72.42539 | 1 |
| NCT03364309 | Risankizumab VS Placebo | 2.6071429 | 0.456201 | 14.89955 | 1 |
| NCT03364309 | Secukinumab VS Placebo | 33.5 | 2.535645 | 442.5895 | 1 |
| NCT03364309 | Ustekinumab VS Placebo | 19.433888 | 3.182274 | 118.6812 | 1 |
| NCT03370133 | Bimekizumab VS Placebo | 31.85 | 5.019929 | 202.0791 | 1 |
| NCT03370133 | Guselkumab VS Placebo | 10.246258 | 3.745573 | 28.0293 | 1 |
| NCT03370133 | Ixekizumab VS Placebo | 14.33099 | 4.045927 | 50.76149 | 1 |
| NCT03370133 | Risankizumab VS Placebo | 2.6071429 | 0.505694 | 13.44132 | 1 |
| NCT03370133 | Secukinumab VS Placebo | 33.5 | 2.715175 | 413.3251 | 1 |
| NCT03410992 | Bimekizumab VS Placebo | 13.357143 | 2.280362 | 78.23902 | 1 |
| NCT03410992 | Guselkumab VS Placebo | 10.246258 | 3.745573 | 28.02931 | 1 |
| NCT03410992 | Ixekizumab VS Placebo | 14.33099 | 4.045927 | 50.76149 | 1 |
| NCT03410992 | Risankizumab VS Placebo | 2.6071429 | 0.505694 | 13.44132 | 1 |
| NCT03410992 | Secukinumab VS Placebo | 33.5 | 2.715174 | 413.3252 | 1 |
| NCT03410992 | Ustekinumab VS Placebo | 15.321429 | 2.380868 | 98.59688 | 1 |
| NCT03998683 | Bimekizumab VS Placebo | 19.487167 | 9.325792 | 40.72036 | 1 |
| NCT03998683 | Guselkumab VS Placebo | 18.836057 | 9.9942 | 35.5003 | 1 |
| NCT03998683 | Ixekizumab VS Placebo | 13.735246 | 6.863265 | 27.48794 | 1 |
| NCT03998683 | Risankizumab VS Placebo | 2.6071429 | 1.262614 | 5.383431 | 1 |
| NCT03998683 | Secukinumab VS Placebo | 33.5 | 4.368934 | 256.8704 | 1 |
| NCT03998683 | Ustekinumab VS Placebo | 20.362001 | 7.253717 | 57.15843 | 1 |
| NCT04713592 | Bimekizumab VS Placebo | 20.178319 | 6.108919 | 66.65084 | 1 |
| NCT04713592 | Guselkumab VS Placebo | 10.284711 | 4.032978 | 26.22759 | 1 |
| NCT04713592 | Ixekizumab VS Placebo | 14.28766 | 4.389424 | 46.50661 | 1 |
| NCT04713592 | Risankizumab VS Placebo | 33.5 | 2.948944 | 380.5599 | 1 |
| NCT04713592 | Secukinumab VS Placebo | 19.539642 | 3.882907 | 98.32778 | 1 |
| UNCOVER_1_2_3 | Bimekizumab VS Placebo | 20.271111 | 5.286046 | 77.73635 | 1 |
| UNCOVER_1_2_3 | Guselkumab VS Placebo | 10.221024 | 3.532505 | 29.57372 | 1 |
| UNCOVER_1_2_3 | Ixekizumab VS Placebo | 17.916667 | 2.477448 | 129.5716 | 1 |
| UNCOVER_1_2_3 | Risankizumab VS Placebo | 2.6071429 | 0.456201 | 14.89955 | 1 |
| UNCOVER_1_2_3 | Secukinumab VS Placebo | 33.5 | 2.535645 | 442.5895 | 1 |
| UNCOVER_1_2_3 | Ustekinumab VS Placebo | 19.433888 | 3.182274 | 118.6812 | 1 |

*Meta-Regression Analysis*

| **Table S9. Meta-Regression Analysis of Age for Complete Clearance in Palmoplantar Psoriasis** | | | | | | | |
| --- | --- | --- | --- | --- | --- | --- | --- |
| **Interventions** | **Covariates** | **Coefficient** | **Standard Error** | **Z-statistic** | **P>z** | **lower confidence interval** | **upper confidence interval** |
| Guselkumab VS Bimekizumab |  |  |  |  |  |  |  |
|  | Age | 0.2424774 | 0.4283757 | 0.57 | 0.571 | -0.5971236 | 1.082078 |
|  | _cons | -11.37928 | 19.42937 | -0.59 | 0.558 | -49.46016 | 26.70159 |
| Ixekizumab VS Bimekizumab |  |  |  |  |  |  |  |
|  | Age | 0.4239236 | 0.4351558 | 0.97 | 0.33 | -0.428966 | 1.276813 |
|  | _cons | -19.47158 | 19.76864 | -0.98 | 0.325 | -58.2174 | 19.27424 |
| Bimekizumab VS Placebo |  |  |  |  |  |  |  |
|  | Age | 0.4827506 | 0.4214476 | 1.15 | 0.252 | -0.3432715 | 1.308773 |
|  | _cons | -24.84687 | 19.10271 | -1.3 | 0.193 | -62.28749 | 12.59376 |
| Risankizumab VS Bimekizumab |  |  |  |  |  |  |  |
|  | _cons | 3.579897 | 4.909775 | 0.73 | 0.466 | -6.043086 | 13.20288 |
| Secukinumab VS Bimekizumab |  |  |  |  |  |  |  |
|  | _cons | 2.753931 | 2.225116 | 1.24 | 0.216 | -1.60722 | 7.115078 |
| Ustekinumab VS Bimekizumab |  |  |  |  |  |  |  |
|  | _cons | 0.1371977 | 0.4608764 | 0.3 | 0.766 | -0.7661036 | 1.040499 |

| **Table S10. Meta-Regression Analysis of Previous Bio Therapy for Complete Clearance in Palmoplantar Psoriasis** | | | | | | | |
| --- | --- | --- | --- | --- | --- | --- | --- |
| **Interventions** | **Covariates** | **Coefficient** | **Standard Error** | **Z-statistic** | **P>z** | **lower confidence interval** | **upper confidence interval** |
| Guselkumab VS Bimekizumab |  |  |  |  |  |  |  |
|  | Previous Bio Therapy | -0.0844349 | 0.1543631 | -0.55 | 0.584 | -0.386981 | 0.2181111 |
|  | _cons | 5.253497 | 6.276852 | 0.84 | 0.403 | -7.048908 | 17.5559 |
| Ixekizumab VS Bimekizumab |  |  |  |  |  |  |  |
|  | Previous Bio Therapy | -0.1899548 | 0.1545871 | -1.23 | 0.219 | -0.4929399 | 0.1130303 |
|  | _cons | 7.233978 | 6.316308 | 1.15 | 0.252 | -5.145759 | 19.61371 |
| Bimekizumab VS Placebo |  |  |  |  |  |  |  |
|  | Previous Bio Therapy | -0.1737658 | 0.1516839 | -1.15 | 0.252 | -0.4710608 | 0.1235292 |
|  | _cons | 4.184739 | 6.25662 | 0.67 | 0.504 | -8.07801 | 16.44749 |
| Risankizumab VS Bimekizumab |  |  |  |  |  |  |  |
|  | _cons | 3.248947 | 4.622152 | 0.7 | 0.482 | -5.810305 | 12.3082 |
| Secukinumab VS Bimekizumab |  |  |  |  |  |  |  |
|  | _cons | 6.045502 | 4.929796 | 1.23 | 0.22 | -3.61672 | 15.70772 |
| Ustekinumab VS Bimekizumab |  |  |  |  |  |  |  |
|  | _cons | 0.1371827 | 0.4608727 | 0.3 | 0.766 | -0.7661112 | 1.040477 |

| **Table S11. Meta-Regression Analysis of Disease Duration for Complete Clearance in Palmoplantar Psoriasis** | | | | | | | |
| --- | --- | --- | --- | --- | --- | --- | --- |
| **Interventions** | **Covariates** | **Coefficient** | **Standard Error** | **Z-statistic** | **P>z** | **lower confidence interval** | **upper confidence interval** |
| Guselkumab VS Bimekizumab |  |  |  |  |  |  |  |
|  | Disease Duration | -0.0065749 | 0.3359079 | -0.02 | 0.984 | -0.6649422 | 0.6517925 |
|  | _cons | 0.1872571 | 6.045261 | 0.03 | 0.975 | -11.66124 | 12.03575 |
| Ixekizumab VS Bimekizumab |  |  |  |  |  |  |  |
|  | Disease Duration | -0.4922114 | 0.3966627 | -1.24 | 0.215 | -1.269656 | 0.2852332 |
|  | _cons | 8.605081 | 7.242619 | 1.19 | 0.235 | -5.590191 | 22.80035 |
| Bimekizumab VS Placebo |  |  |  |  |  |  |  |
|  | Disease Duration | -0.3620737 | 0.3160444 | -1.15 | 0.252 | -0.9815094 | 0.2573619 |
|  | _cons | 3.599406 | 5.746357 | 0.63 | 0.531 | -7.663247 | 14.86206 |
| Risankizumab VS Bimekizumab |  |  |  |  |  |  |  |
|  | _cons | 0.9369239 | 2.627097 | 0.36 | 0.721 | -4.212092 | 6.085939 |
| Secukinumab VS Bimekizumab |  |  |  |  |  |  |  |
|  | _cons | 3.526418 | 2.829944 | 1.25 | 0.213 | -2.020171 | 9.073007 |
| Ustekinumab VS Bimekizumab |  |  |  |  |  |  |  |
|  | _cons | 0.1372003 | 0.4608746 | 0.3 | 0.766 | -0.7660974 | 1.040498 |

| **Table S12. Meta-Regression Analysis of Baseline PASI for Complete Clearance in Palmoplantar Psoriasis** | | | | | | | |
| --- | --- | --- | --- | --- | --- | --- | --- |
| **Interventions** | **Covariates** | **Coefficient** | **Standard Error** | **Z-statistic** | **P>z** | **lower confidence interval** | **upper confidence interval** |
| Guselkumab VS Bimekizumab |  |  |  |  |  |  |  |
|  | Baseline PASI | 0.8438761 | 0.6330967 | 1.33 | 0.183 | -0.3969706 | 2.084723 |
|  | _cons | -17.82007 | 13.28132 | -1.34 | 0.18 | -43.85098 | 8.210842 |
| Ixekizumab VS Bimekizumab |  |  |  |  |  |  |  |
|  | Baseline PASI | 0.9450164 | 0.7518446 | 1.26 | 0.209 | -0.528572 | 2.418605 |
|  | _cons | -20.84926 | 16.48686 | -1.26 | 0.206 | -53.16291 | 11.46439 |
| Bimekizumab VS Placebo |  |  |  |  |  |  |  |
|  | Baseline PASI | 0.7238538 | 0.6319297 | 1.15 | 0.252 | -0.5147058 | 1.962413 |
|  | _cons | -18.15506 | 13.26221 | -1.37 | 0.171 | -44.14852 | 7.838406 |
| Risankizumab VS Bimekizumab |  |  |  |  |  |  |  |
|  | _cons | -8.727711 | 5.886983 | -1.48 | 0.138 | -20.26599 | 2.810564 |
| Secukinumab VS Bimekizumab |  |  |  |  |  |  |  |
|  | _cons | -8.925052 | 8.338199 | -1.07 | 0.284 | -25.26762 | 7.417519 |
| Ustekinumab VS Bimekizumab |  |  |  |  |  |  |  |
|  | _cons | 0.1371643 | 0.4608713 | 0.3 | 0.766 | -0.7661268 | 1.040455 |

| **Table S13. Meta-Regression Analysis of Timepoint for Complete Clearance in Palmoplantar Psoriasis** | | | | | | | |
| --- | --- | --- | --- | --- | --- | --- | --- |
| **Interventions** | **Covariates** | **Coefficient** | **Standard Error** | **Z-statistic** | **P>z** | **lower confidence interval** | **upper confidence interval** |
| Guselkumab VS Bimekizumab |  |  |  |  |  |  |  |
|  | _cons | -0.6739432 | 0.7748214 | -0.87 | 0.384 | -2.192565 | 0.8446789 |
| Ixekizumab VS Bimekizumab |  |  |  |  |  |  |  |
|  | _cons | 0.90493 | 332.0204 | 0 | 0.998 | -649.8431 | 651.653 |
| Bimekizumab VS Placebo |  |  |  |  |  |  |  |
|  | Timepoint | -0.3125342 | 83.00506 | 0 | 0.997 | -162.9995 | 162.3744 |
|  | _cons | 1.995943 | 1328.081 | 0 | 0.999 | -2600.994 | 2604.986 |
| Risankizumab VS Bimekizumab |  |  |  |  |  |  |  |
|  | _cons | -2.046346 | 0.9826249 | -2.08 | 0.037 | -3.972256 | -0.1204371 |
| Secukinumab VS Bimekizumab |  |  |  |  |  |  |  |
|  | _cons | 0.5069429 | 1.381608 | 0.37 | 0.714 | -2.200959 | 3.214844 |
| Ustekinumab VS Bimekizumab |  |  |  |  |  |  |  |
|  | _cons | -0.0321608 | 0.7835214 | -0.04 | 0.967 | -1.567834 | 1.503513 |

***Supplement 3 Nail Psoriasis***

| **Table S14.** Node-Splitting Analysis of Inconsistency for Nail Psoriasis | | | | | |
| --- | --- | --- | --- | --- | --- |
| Comparison (Side) | Direct Evidence Coef. (SE) | Indirect Evidence Coef. (SE) | | Inconsistency Factor (Difference) Coef. (SE) | *P*-value |
| Bimekizumab vs. Placebo | -1.83 (0.52) | | -0.34 (1.67) | -1.50 (1.82) | 0.411 |
| Bimekizumab vs. Ustekinumab | -0.96 (0.54) | | -2.06 (0.97) | 1.10 (1.12) | 0.326 |
| Brodalumab vs. Ustekinumab | -1.01 (0.55) | | -2.56 (1267.74) | 1.55 (1267.74) | 0.999 |
| Guselkumab vs. Ixekizumab | 0.74 (0.33) | | -0.22 (0.38) | 0.96 (0.50) | 0.056 |
| Guselkumab vs. Placebo | -1.56 (0.29) | | -0.60 (0.41) | -0.96 (0.50) | 0.056 |
| Ixekizumab vs. Placebo | -1.25 (0.21) | | -2.30 (0.27) | 1.04 (0.33) | **0.001**† |
| Ixekizumab vs. Ustekinumab | -1.40 (0.55) | | -0.65 (0.73) | -0.75 (0.91) | 0.411 |

SE: Standard Error.

† Significant Inconsistency: The comparison between Ixekizumab and Placebo shows a P-value < 0.05, indicating significant inconsistency between direct and indirect evidence sources.

Table S15 GRADE Certainty of Evidence Profile for Nail Psoriasis Outcomes

| **Comparison** | **Number of studies** | **Within-study bias** | **Reporting bias** | **Indirectness** | **Imprecision** | **Heterogeneity** | **Incoherence** | **Confidence rating** | **Reason(s) for downgrading** |
| --- | --- | --- | --- | --- | --- | --- | --- | --- | --- |
| Bimekizumab:Placebo | 2 | No concerns | Low risk | No concerns | No concerns | Some concerns | No concerns | Moderate | ["Heterogeneity"] |
| Bimekizumab:Ustekinumab | 1 | No concerns | Low risk | No concerns | No concerns | Some concerns | No concerns | Moderate | ["Heterogeneity"] |
| Brodalumab:Ustekinumab | 1 | Some concerns | Low risk | No concerns | Some concerns | Some concerns | Some concerns | Very low | ["Within-study bias","Imprecision","Heterogeneity","Incoherence"] |
| Guselkumab:Ixekizumab | 1 | No concerns | Low risk | No concerns | Some concerns | No concerns | No concerns | Moderate | ["Imprecision"] |
| Guselkumab:Placebo | 2 | No concerns | Low risk | No concerns | No concerns | Some concerns | No concerns | Moderate | ["Heterogeneity"] |
| Ixekizumab:Placebo | 2 | No concerns | Low risk | No concerns | No concerns | No concerns | No concerns | High | [] |
| Ixekizumab:Ustekinumab | 1 | Some concerns | Low risk | No concerns | No concerns | Some concerns | No concerns | Low | ["Within-study bias","Heterogeneity"] |
| Placebo:Risankizumab | 1 | Some concerns | Low risk | No concerns | No concerns | Some concerns | Some concerns | Very low | ["Within-study bias","Heterogeneity",  "Incoherence"] |
| Placebo:Ustekinumab | 1 | No concerns | Low risk | No concerns | Some concerns | No concerns | No concerns | Moderate | ["Imprecision"] |
| Bimekizumab:Brodalumab | 0 | No concerns | Low risk | No concerns | Some concerns | No concerns | Some concerns | Low | ["Imprecision",  "Incoherence"] |
| Bimekizumab:Guselkumab | 0 | No concerns | Low risk | No concerns | Some concerns | No concerns | Some concerns | Low | ["Imprecision",  "Incoherence"] |
| Bimekizumab:Ixekizumab | 0 | No concerns | Low risk | No concerns | Some concerns | No concerns | Some concerns | Low | ["Imprecision",  "Incoherence"] |
| Bimekizumab:Risankizumab | 0 | No concerns | Low risk | No concerns | Some concerns | No concerns | Some concerns | Low | ["Imprecision",  "Incoherence"] |
| Brodalumab:Guselkumab | 0 | No concerns | Low risk | No concerns | Some concerns | No concerns | Some concerns | Low | ["Imprecision",  "Incoherence"] |
| Brodalumab:Ixekizumab | 0 | Some concerns | Low risk | No concerns | Some concerns | No concerns | Some concerns | Very low | ["Within-study bias","Imprecision",  "Incoherence"] |
| Brodalumab:Placebo | 0 | Some concerns | Low risk | No concerns | No concerns | Some concerns | Some concerns | Very low | ["Within-study bias","Heterogeneity",  "Incoherence"] |
| Brodalumab:Risankizumab | 0 | Some concerns | Low risk | No concerns | Some concerns | No concerns | Some concerns | Very low | ["Within-study bias","Imprecision",  "Incoherence"] |
| Guselkumab:Risankizumab | 0 | Some concerns | Low risk | No concerns | Some concerns | No concerns | Some concerns | Very low | ["Within-study bias","Imprecision",  "Incoherence"] |
| Guselkumab:Ustekinumab | 0 | No concerns | Low risk | No concerns | Some concerns | Some concerns | Some concerns | Very low | ["Imprecision","Heterogeneity","Incoherence"] |
| Ixekizumab:Risankizumab | 0 | Some concerns | Low risk | No concerns | Some concerns | No concerns | Some concerns | Very low | ["Within-study bias","Imprecision",  "Incoherence"] |
| Risankizumab:Ustekinumab | 0 | Some concerns | Low risk | No concerns | Some concerns | Some concerns | Some concerns | Very low | ["Within-study bias","Imprecision","Heterogeneity","Incoherence"] |

| **Table S16. Sensitivity analysis results for complete clearance in nail psoriasis** | | | | | |
| --- | --- | --- | --- | --- | --- |
| **dropped_id** | **comparison** | **effect size** | **lower confidence interval** | **upper confidence interval** | **Connected Network Status** |
| AMAGINE_2_3 | Bimekizumab VS Placebo | 5.1963435 | 2.097775 | 12.87173 | 1 |
| AMAGINE_2_3 | Guselkumab VS Placebo | 3.5445546 | 1.913677 | 6.565301 | 1 |
| AMAGINE_2_3 | Ixekizumab VS Placebo | 4.763817 | 2.690292 | 8.435499 | 1 |
| AMAGINE_2_3 | Risankizumab VS Placebo | 2.8478702 | 1.080319 | 7.50738 | 1 |
| AMAGINE_2_3 | Ustekinumab VS Placebo | 1.5346518 | 0.642692 | 3.664519 | 1 |
| NCT01695239 | Bimekizumab VS Placebo | 5.4820479 | 2.284693 | 13.154 | 1 |
| NCT01695239 | Brodalumab VS Placebo | 4.7772612 | 1.27633 | 17.88112 | 1 |
| NCT01695239 | Guselkumab VS Placebo | 3.8501867 | 2.128621 | 6.964103 | 1 |
| NCT01695239 | Ixekizumab VS Placebo | 5.9370512 | 3.197057 | 11.02532 | 1 |
| NCT01695239 | Risankizumab VS Placebo | 2.8478702 | 1.153849 | 7.028967 | 1 |
| NCT01695239 | Ustekinumab VS Placebo | 1.7412836 | 0.743828 | 4.076305 | 1 |
| NCT02207231 | Bimekizumab VS Placebo | 5.3135316 | 1.984398 | 14.2278 | 1 |
| NCT02207231 | Brodalumab VS Placebo | 4.2632165 | 0.919477 | 19.76668 | 1 |
| NCT02207231 | Guselkumab VS Placebo | 3.6597375 | 1.55127 | 8.634008 | 1 |
| NCT02207231 | Ixekizumab VS Placebo | 4.7826786 | 2.455746 | 9.314487 | 1 |
| NCT02207231 | Risankizumab VS Placebo | 2.8478703 | 0.942592 | 8.604318 | 1 |
| NCT02207231 | Ustekinumab VS Placebo | 1.5539174 | 0.592957 | 4.072232 | 1 |
| NCT02207244 | Bimekizumab VS Placebo | 4.7055148 | 2.129667 | 10.39687 | 1 |
| NCT02207244 | Brodalumab VS Placebo | 3.7960316 | 1.257774 | 11.45664 | 1 |
| NCT02207244 | Guselkumab VS Placebo | 2.4513336 | 1.353159 | 4.440747 | 1 |
| NCT02207244 | Ixekizumab VS Placebo | 4.2466866 | 2.730493 | 6.604796 | 1 |
| NCT02207244 | Risankizumab VS Placebo | 2.8478702 | 1.404047 | 5.776421 | 1 |
| NCT02207244 | Ustekinumab VS Placebo | 1.3836309 | 0.673831 | 2.841121 | 1 |
| NCT02561806 | Bimekizumab VS Placebo | 6.2639354 | 2.266382 | 17.31256 | 1 |
| NCT02561806 | Brodalumab VS Placebo | 6.1888183 | 1.145372 | 33.44021 | 1 |
| NCT02561806 | Guselkumab VS Placebo | 3.4249033 | 1.800249 | 6.515746 | 1 |
| NCT02561806 | Ixekizumab VS Placebo | 4.3316857 | 2.294426 | 8.177864 | 1 |
| NCT02561806 | Risankizumab VS Placebo | 2.8478702 | 1.041598 | 7.786461 | 1 |
| NCT02561806 | Ustekinumab VS Placebo | 2.2557882 | 0.628765 | 8.092978 | 1 |
| NCT03370133 | Bimekizumab VS Placebo | 8.3479319 | 1.792207 | 38.88387 | 1 |
| NCT03370133 | Brodalumab VS Placebo | 2.9096459 | 0.499579 | 16.94635 | 1 |
| NCT03370133 | Guselkumab VS Placebo | 3.4324721 | 1.735299 | 6.789531 | 1 |
| NCT03370133 | Ixekizumab VS Placebo | 4.3084822 | 2.192146 | 8.467966 | 1 |
| NCT03370133 | Risankizumab VS Placebo | 2.8478702 | 0.975278 | 8.315954 | 1 |
| NCT03370133 | Ustekinumab VS Placebo | 1.0605487 | 0.282067 | 3.987573 | 1 |
| NCT03410992 | Bimekizumab VS Placebo | 3.9159918 | 1.234649 | 12.42053 | 1 |
| NCT03410992 | Brodalumab VS Placebo | 3.7095559 | 0.886047 | 15.53056 | 1 |
| NCT03410992 | Guselkumab VS Placebo | 3.5030331 | 1.871552 | 6.556718 | 1 |
| NCT03410992 | Ixekizumab VS Placebo | 4.6084203 | 2.564279 | 8.28207 | 1 |
| NCT03410992 | Risankizumab VS Placebo | 2.8478704 | 1.062876 | 7.630587 | 1 |
| NCT03410992 | Ustekinumab VS Placebo | 1.3521111 | 0.530152 | 3.448452 | 1 |
| NCT03573323 | Bimekizumab VS Placebo | 4.5294641 | 2.008158 | 10.21635 | 1 |
| NCT03573323 | Brodalumab VS Placebo | 3.5378892 | 1.154591 | 10.84078 | 1 |
| NCT03573323 | Guselkumab VS Placebo | 4.7525457 | 2.691391 | 8.3922 | 1 |
| NCT03573323 | Ixekizumab VS Placebo | 3.8021893 | 2.331554 | 6.200432 | 1 |
| NCT03573323 | Risankizumab VS Placebo | 2.8478702 | 1.399212 | 5.796381 | 1 |
| NCT03573323 | Ustekinumab VS Placebo | 1.2895396 | 0.615707 | 2.700817 | 1 |
| NCT03675308 | Bimekizumab VS Placebo | 5.1963423 | 2.097775 | 12.87172 | 1 |
| NCT03675308 | Brodalumab VS Placebo | 4.2103591 | 1.061115 | 16.70613 | 1 |
| NCT03675308 | Guselkumab VS Placebo | 3.5445544 | 1.913678 | 6.5653 | 1 |
| NCT03675308 | Ixekizumab VS Placebo | 4.7638165 | 2.690292 | 8.435497 | 1 |
| NCT03675308 | Risankizumab VS Placebo | 1.5346511 | 0.642692 | 3.664515 | 1 |
| UNCOVER_1_2_3 | Bimekizumab VS Placebo | 5.4516386 | 2.07722 | 14.30776 | 1 |
| UNCOVER_1_2_3 | Brodalumab VS Placebo | 4.5551471 | 1.008992 | 20.56445 | 1 |
| UNCOVER_1_2_3 | Guselkumab VS Placebo | 3.7366307 | 1.865698 | 7.483746 | 1 |
| UNCOVER_1_2_3 | Ixekizumab VS Placebo | 5.3930015 | 2.440286 | 11.91846 | 1 |
| UNCOVER_1_2_3 | Risankizumab VS Placebo | 2.8478702 | 0.984435 | 8.238597 | 1 |
| UNCOVER_1_2_3 | Ustekinumab VS Placebo | 1.6603244 | 0.629297 | 4.380569 | 1 |

***Meta-Regression Analysis***

| **Table S17. Meta-Regression Analysis of Age for Complete Clearance in Nail Psoriasis** | | | | | | | |
| --- | --- | --- | --- | --- | --- | --- | --- |
| **Interventions** | **Covariates** | **Coefficient** | **Standard Error** | **Z-statistic** | **P>z** | **lower confidence interval** | **upper confidence interval** |
| Brodalumab VS Bimekizumab |  |  |  |  |  |  |  |
|  | _cons | -0.4803107 | 0.568838 | -0.84 | 0.398 | -1.595213 | 0.6345913 |
| Guselkumab VS Bimekizumab |  |  |  |  |  |  |  |
|  | Age | 0.03328 | 0.4615707 | 0.07 | 0.943 | -0.871382 | 0.937942 |
|  | _cons | -2.123213 | 20.91677 | -0.1 | 0.919 | -43.11934 | 38.87291 |
| Ixekizumab VS Bimekizumab |  |  |  |  |  |  |  |
|  | Age | 0.1994366 | 0.4630104 | 0.43 | 0.667 | -0.7080472 | 1.10692 |
|  | _cons | -9.51602 | 20.9966 | -0.45 | 0.65 | -50.6686 | 31.63656 |
| Placebo VS Bimekizumab |  |  |  |  |  |  |  |
|  | Age | 0.2972677 | 0.454946 | 0.65 | 0.513 | -0.5944101 | 1.188945 |
|  | _cons | -15.29096 | 20.61599 | -0.74 | 0.458 | -55.69757 | 25.11564 |
| Risankizumab VS Bimekizumab |  |  |  |  |  |  |  |
|  | _cons | 1.005438 | 2.767504 | 0.36 | 0.716 | -4.41877 | 6.429646 |
| Ustekinumab VS Bimekizumab |  |  |  |  |  |  |  |
|  | Age | 0.4829471 | 0.4103272 | 1.18 | 0.239 | -0.3212794 | 1.287174 |
|  | _cons | -23.22217 | 18.81516 | -1.23 | 0.217 | -60.09921 | 13.65486 |

| **Table S18. Meta-Regression Analysis of Previous Bio Therapy for Complete Clearance in Nail Psoriasis** | | | | | | | |
| --- | --- | --- | --- | --- | --- | --- | --- |
| **Interventions** | **Covariates** | **Coefficient** | **Standard Error** | **Z-statistic** | **P>z** | **lower confidence interval** | **upper confidence interval** |
| Brodalumab VS Bimekizumab |  |  |  |  |  |  |  |
|  | _cons | 0.8889468 | 2.525619 | 0.35 | 0.725 | -4.061176 | 5.839069 |
| Guselkumab VS Bimekizumab |  |  |  |  |  |  |  |
|  | Previous Bio Therapy | -0.294005 | 0.2204442 | -1.33 | 0.182 | -0.7260676 | 0.1380577 |
|  | _cons | 8.049454 | 8.149782 | 0.99 | 0.323 | -7.923825 | 24.02273 |
| Ixekizumab VS Bimekizumab |  |  |  |  |  |  |  |
|  | Previous Bio Therapy | -0.090136 | 0.1879988 | -0.48 | 0.632 | -0.4586068 | 0.2783348 |
|  | _cons | 3.486597 | 7.729127 | 0.45 | 0.652 | -11.66221 | 18.63541 |
| Placebo VS Bimekizumab |  |  |  |  |  |  |  |
|  | Previous Bio Therapy | -0.1069901 | 0.18655 | -0.57 | 0.566 | -0.4726213 | 0.2586411 |
|  | _cons | 2.585621 | 7.713218 | 0.34 | 0.737 | -12.53201 | 17.70325 |
| Risankizumab VS Bimekizumab |  |  |  |  |  |  |  |
|  | _cons | 3.632192 | 7.723431 | 0.47 | 0.638 | -11.50545 | 18.76984 |
| Ustekinumab VS Bimekizumab |  |  |  |  |  |  |  |
|  | Previous Bio Therapy | -0.0710195 | 0.2029787 | -0.35 | 0.726 | -0.4688503 | 0.3268114 |
|  | _cons | 1.811431 | 7.976716 | 0.23 | 0.82 | -13.82265 | 17.44551 |

| **Table S19. Meta-Regression Analysis of Disease Duration for Complete Clearance in Nail Psoriasis** | | | | | | | |
| --- | --- | --- | --- | --- | --- | --- | --- |
| **Interventions** | **Covariates** | **Coefficient** | **Standard Error** | **Z-statistic** | **P>z** | **lower confidence interval** | **upper confidence interval** |
| Brodalumab VS Bimekizumab |  |  |  |  |  |  |  |
|  | _cons | -1.939898 | 1.167256 | -1.66 | 0.097 | -4.227678 | 0.3478825 |
| Guselkumab VS Bimekizumab |  |  |  |  |  |  |  |
|  | Disease Duration | 1.219184 | 0.6091601 | 2 | 0.045 | 0.025252 | 2.413116 |
|  | _cons | -21.94261 | 10.85213 | -2.02 | 0.043 | -43.21239 | -0.6728321 |
| Ixekizumab VS Bimekizumab |  |  |  |  |  |  |  |
|  | Disease Duration | -0.0174029 | 0.4070628 | -0.04 | 0.966 | -0.8152315 | 0.7804256 |
|  | _cons | -0.2948737 | 7.416584 | -0.04 | 0.968 | -14.83111 | 14.24136 |
| Placebo VS Bimekizumab |  |  |  |  |  |  |  |
|  | Disease Duration | -0.2229518 | 0.3520076 | -0.63 | 0.526 | -0.912874 | 0.4669705 |
|  | _cons | 2.225552 | 6.408465 | 0.35 | 0.728 | -10.33481 | 14.78591 |
| Risankizumab VS Bimekizumab |  |  |  |  |  |  |  |
|  | _cons | 1.689166 | 3.928116 | 0.43 | 0.667 | -6.009799 | 9.388132 |
| Ustekinumab VS Bimekizumab |  |  |  |  |  |  |  |
|  | Disease Duration | -1.053348 | 0.6469128 | -1.63 | 0.103 | -2.321274 | 0.2145774 |
|  | _cons | 17.05394 | 11.2251 | 1.52 | 0.129 | -4.946851 | 39.05473 |

| **Table S20. Meta-Regression Analysis of** **Baseline PASI for Complete Clearance in Nail Psoriasis** | | | | | | | |
| --- | --- | --- | --- | --- | --- | --- | --- |
| **Interventions** | **Covariates** | **Coefficient** | **Standard Error** | **Z-statistic** | **P>z** | **lower confidence interval** | **upper confidence interval** |
| Brodalumab VS Bimekizumab |  |  |  |  |  |  |  |
|  | _cons | -1.072643 | 0.8624845 | -1.24 | 0.214 | -2.763081 | 0.6177959 |
| Guselkumab VS Bimekizumab |  |  |  |  |  |  |  |
|  | Baseline PASI | 0.9006817 | 0.7305588 | 1.23 | 0.218 | -0.5311873 | 2.332551 |
|  | _cons | -19.44292 | 15.32018 | -1.27 | 0.204 | -49.46992 | 10.58409 |
| Ixekizumab VS Bimekizumab |  |  |  |  |  |  |  |
|  | Baseline PASI | 0.4764171 | 0.7086875 | 0.67 | 0.501 | -0.9125849 | 1.865419 |
|  | _cons | -10.45886 | 14.85951 | -0.7 | 0.482 | -39.58297 | 18.66524 |
| Placebo VS Bimekizumab |  |  |  |  |  |  |  |
|  | Baseline PASI | 0.4457188 | 0.7078718 | 0.63 | 0.529 | -0.9416844 | 1.833122 |
|  | _cons | -11.16997 | 14.8473 | -0.75 | 0.452 | -40.27014 | 17.9302 |
| Risankizumab VS Bimekizumab |  |  |  |  |  |  |  |
|  | _cons | -5.456724 | 7.447456 | -0.73 | 0.464 | -20.05347 | 9.140021 |
| Ustekinumab VS Bimekizumab |  |  |  |  |  |  |  |
|  | Baseline PASI | 0.8642793 | 0.6431138 | 1.34 | 0.179 | -0.3962006 | 2.124759 |
|  | _cons | -19.54033 | 13.70045 | -1.43 | 0.154 | -46.39272 | 7.312056 |

| **Table S21. Meta-Regression Analysis of** **Timepoint for Complete Clearance in Nail Psoriasis** | | | | | | | |
| --- | --- | --- | --- | --- | --- | --- | --- |
| **Interventions** | **Covariates** | **Coefficient** | **Standard Error** | **Z-statistic** | **P>z** | **lower confidence interval** | **upper confidence interval** |
| Brodalumab VS Bimekizumab |  |  |  |  |  |  |  |
|  | _cons | 0.4893178 | 475.2366 | 0 | 0.999 | -930.9573 | 931.936 |
| Guselkumab VS Bimekizumab |  |  |  |  |  |  |  |
|  | Timepoint | 0.3172253 | 13.20163 | 0.02 | 0.981 | -25.55749 | 26.19194 |
|  | _cons | -5.342445 | 211.2257 | -0.03 | 0.98 | -419.3373 | 408.6524 |
| Ixekizumab VS Bimekizumab |  |  |  |  |  |  |  |
|  | Timepoint | 0.041997 | 13.20103 | 0 | 0.997 | -25.83154 | 25.91554 |
|  | _cons | -1.301972 | 211.2177 | -0.01 | 0.995 | -415.2811 | 412.6772 |
| Placebo VS Bimekizumab |  |  |  |  |  |  |  |
|  | Timepoint | 0.0790761 | 13.20111 | 0.01 | 0.995 | -25.79462 | 25.95277 |
|  | _cons | -3.092848 | 211.2187 | -0.01 | 0.988 | -417.0739 | 410.8882 |
| Risankizumab VS Bimekizumab |  |  |  |  |  |  |  |
|  | _cons | -0.14845 | 105.6098 | 0 | 0.999 | -207.1399 | 206.843 |
| Ustekinumab VS Bimekizumab |  |  |  |  |  |  |  |
|  | Timepoint | 0.0132095 | 13.20102 | 0 | 0.999 | -25.86032 | 25.88674 |
|  | _cons | -1.206821 | 211.2172 | -0.01 | 0.995 | -415.1848 | 412.7712 |

***Supplement 4	Scalp Psoriasis***

**Table S22. GRADE Certainty of Evidence Profile for Scalp Psoriasis Outcomes**

| **Comparison** | **Number of studies** | **Within-study bias** | **Reporting bias** | **Indirectness** | **Imprecision** | **Heterogeneity** | **Incoherence** | **Confidence rating** | **Reason(s) for downgrading** |
| --- | --- | --- | --- | --- | --- | --- | --- | --- | --- |
| Bimekizumab:Placebo | 1 | No concerns | Low risk | No concerns | No concerns | No concerns | No concerns | High | [] |
| Bimekizumab:Ustekinumab | 1 | No concerns | Low risk | No concerns | No concerns | No concerns | No concerns | High | [] |
| Brodalumab:Placebo | 1 | Some concerns | Low risk | No concerns | No concerns | No concerns | No concerns | Moderate | ["Within-study bias"] |
| Guselkumab:Placebo | 5 | No concerns | Low risk | No concerns | No concerns | No concerns | No concerns | High | [] |
| Ixekizumab:Placebo | 2 | Some concerns | Low risk | No concerns | No concerns | No concerns | No concerns | Moderate | ["Within-study bias"] |
| Placebo:Risankizumab | 1 | No concerns | Low risk | No concerns | No concerns | No concerns | No concerns | High | [] |
| Placebo:Secukinumab | 1 | No concerns | Low risk | No concerns | No concerns | No concerns | No concerns | High | [] |
| Placebo:Tildrakizumab | 1 | No concerns | Low risk | No concerns | No concerns | No concerns | No concerns | High | [] |
| Placebo:Ustekinumab | 1 | No concerns | Low risk | No concerns | No concerns | No concerns | No concerns | High | [] |
| Bimekizumab:Brodalumab | 0 | No concerns | Low risk | No concerns | Some concerns | No concerns | No concerns | Moderate | ["Imprecision"] |
| Bimekizumab:Guselkumab | 0 | No concerns | Low risk | No concerns | Some concerns | No concerns | No concerns | Moderate | ["Imprecision"] |
| Bimekizumab:Ixekizumab | 0 | No concerns | Low risk | No concerns | Some concerns | No concerns | No concerns | Moderate | ["Imprecision"] |
| Bimekizumab:Risankizumab | 0 | No concerns | Low risk | No concerns | Some concerns | Some concerns | No concerns | Low | ["Imprecision","Heterogeneity"] |
| Bimekizumab:Secukinumab | 0 | No concerns | Low risk | No concerns | Some concerns | No concerns | No concerns | Moderate | ["Imprecision"] |
| Bimekizumab:Tildrakizumab | 0 | No concerns | Low risk | No concerns | Some concerns | No concerns | No concerns | Moderate | ["Imprecision"] |
| Brodalumab:Guselkumab | 0 | No concerns | Low risk | No concerns | Some concerns | No concerns | No concerns | Moderate | ["Imprecision"] |
| Brodalumab:Ixekizumab | 0 | Some concerns | Low risk | No concerns | Some concerns | No concerns | No concerns | Low | ["Within-study bias","Imprecision"] |
| Brodalumab:Risankizumab | 0 | No concerns | Low risk | No concerns | No concerns | Some concerns | No concerns | Moderate | ["Heterogeneity"] |
| Brodalumab:Secukinumab | 0 | No concerns | Low risk | No concerns | Some concerns | No concerns | No concerns | Moderate | ["Imprecision"] |
| Brodalumab:Tildrakizumab | 0 | No concerns | Low risk | No concerns | Some concerns | Some concerns | No concerns | Low | ["Imprecision","Heterogeneity"] |
| Brodalumab:Ustekinumab | 0 | No concerns | Low risk | No concerns | Some concerns | Some concerns | No concerns | Low | ["Imprecision","Heterogeneity"] |
| Guselkumab:Ixekizumab | 0 | No concerns | Low risk | No concerns | Some concerns | No concerns | No concerns | Moderate | ["Imprecision"] |
| Guselkumab:Risankizumab | 0 | No concerns | Low risk | No concerns | Some concerns | Some concerns | No concerns | Low | ["Imprecision","Heterogeneity"] |
| Guselkumab:Secukinumab | 0 | No concerns | Low risk | No concerns | Some concerns | No concerns | No concerns | Moderate | ["Imprecision"] |
| Guselkumab:Tildrakizumab | 0 | No concerns | Low risk | No concerns | Some concerns | Some concerns | No concerns | Low | ["Imprecision","Heterogeneity"] |
| Guselkumab:Ustekinumab | 0 | No concerns | Low risk | No concerns | Some concerns | Some concerns | No concerns | Low | ["Imprecision","Heterogeneity"] |
| Ixekizumab:Risankizumab | 0 | No concerns | Low risk | No concerns | No concerns | No concerns | No concerns | High | [] |
| Ixekizumab:Secukinumab | 0 | No concerns | Low risk | No concerns | Some concerns | No concerns | No concerns | Moderate | ["Imprecision"] |
| Ixekizumab:Tildrakizumab | 0 | No concerns | Low risk | No concerns | No concerns | Some concerns | No concerns | Moderate | ["Heterogeneity"] |
| Ixekizumab:Ustekinumab | 0 | No concerns | Low risk | No concerns | No concerns | No concerns | No concerns | High | [] |
| Risankizumab:Secukinumab | 0 | No concerns | Low risk | No concerns | Some concerns | No concerns | No concerns | Moderate | ["Imprecision"] |
| Risankizumab:Tildrakizumab | 0 | No concerns | Low risk | No concerns | Some concerns | No concerns | No concerns | Moderate | ["Imprecision"] |
| Risankizumab:Ustekinumab | 0 | No concerns | Low risk | No concerns | Some concerns | No concerns | No concerns | Moderate | ["Imprecision"] |
| Secukinumab:Tildrakizumab | 0 | No concerns | Low risk | No concerns | Some concerns | No concerns | No concerns | Moderate | ["Imprecision"] |
| Secukinumab:Ustekinumab | 0 | No concerns | Low risk | No concerns | Some concerns | No concerns | No concerns | Moderate | ["Imprecision"] |
| Tildrakizumab:Ustekinumab | 0 | No concerns | Low risk | No concerns | Some concerns | No concerns | No concerns | Moderate | ["Imprecision"] |

| **Table S23. Sensitivity analysis results for complete clearance in scalp psoriasis** | | | | | |
| --- | --- | --- | --- | --- | --- |
| **dropped_id** | **comparison** | **effect size** | **lower confidence interval** | **upper confidence interval** | **Connected Network Status** |
| NCT01708590 | Bimekizumab VS Placebo | 29.57576 | 14.44436 | 60.55828 | 1 |
| NCT01708590 | Guselkumab VS Placebo | 28.31394 | 20.75561 | 38.62469 | 1 |
| NCT01708590 | Ixekizumab VS Placebo | 42.33392 | 30.7526 | 58.27673 | 1 |
| NCT01708590 | Risankizumab VS Placebo | 10.40714 | 3.933738 | 27.53326 | 1 |
| NCT01708590 | Secukinumab VS Placebo | 21.09091 | 5.797724 | 76.72433 | 1 |
| NCT01708590 | Tildrakizumab VS Placebo | 12.38519 | 4.890323 | 31.3666 | 1 |
| NCT01708590 | Ustekinumab VS Placebo | 13.2833 | 6.375378 | 27.67616 | 1 |
| NCT02207231 | Bimekizumab VS Placebo | 29.57576 | 13.10108 | 66.76745 | 1 |
| NCT02207231 | Brodalumab VS Placebo | 53.15556 | 14.57818 | 193.818 | 1 |
| NCT02207231 | Guselkumab VS Placebo | 27.05882 | 17.12455 | 42.75616 | 1 |
| NCT02207231 | Ixekizumab VS Placebo | 44.06965 | 25.03646 | 77.57225 | 1 |
| NCT02207231 | Risankizumab VS Placebo | 10.40714 | 3.653211 | 29.64751 | 1 |
| NCT02207231 | Secukinumab VS Placebo | 21.09091 | 5.478597 | 81.1935 | 1 |
| NCT02207231 | Tildrakizumab VS Placebo | 12.38519 | 4.527003 | 33.88397 | 1 |
| NCT02207231 | Ustekinumab VS Placebo | 13.2833 | 5.794332 | 30.45148 | 1 |
| NCT02207244 | Bimekizumab VS Placebo | 29.57576 | 14.44436 | 60.55828 | 1 |
| NCT02207244 | Brodalumab VS Placebo | 53.15556 | 15.46578 | 182.6946 | 1 |
| NCT02207244 | Guselkumab VS Placebo | 25.32096 | 17.08874 | 37.51892 | 1 |
| NCT02207244 | Ixekizumab VS Placebo | 42.33392 | 30.7526 | 58.27673 | 1 |
| NCT02207244 | Risankizumab VS Placebo | 10.40714 | 3.933738 | 27.53326 | 1 |
| NCT02207244 | Secukinumab VS Placebo | 21.09091 | 5.797724 | 76.72433 | 1 |
| NCT02207244 | Tildrakizumab VS Placebo | 12.38519 | 4.890323 | 31.3666 | 1 |
| NCT02207244 | Ustekinumab VS Placebo | 13.2833 | 6.375378 | 27.67616 | 1 |
| NCT02267135 | Bimekizumab VS Placebo | 29.57576 | 14.44436 | 60.55828 | 1 |
| NCT02267135 | Brodalumab VS Placebo | 53.15556 | 15.46578 | 182.6946 | 1 |
| NCT02267135 | Guselkumab VS Placebo | 28.31394 | 20.75561 | 38.62468 | 1 |
| NCT02267135 | Ixekizumab VS Placebo | 42.33392 | 30.7526 | 58.27673 | 1 |
| NCT02267135 | Risankizumab VS Placebo | 10.40714 | 3.933738 | 27.53326 | 1 |
| NCT02267135 | Tildrakizumab VS Placebo | 12.38519 | 4.890323 | 31.3666 | 1 |
| NCT02267135 | Ustekinumab VS Placebo | 13.2833 | 6.375378 | 27.67616 | 1 |
| NCT02325219 | Bimekizumab VS Placebo | 29.57576 | 14.44436 | 60.55828 | 1 |
| NCT02325219 | Brodalumab VS Placebo | 53.15556 | 15.46578 | 182.6946 | 1 |
| NCT02325219 | Guselkumab VS Placebo | 27.408 | 19.82158 | 37.89802 | 1 |
| NCT02325219 | Ixekizumab VS Placebo | 42.33392 | 30.7526 | 58.27672 | 1 |
| NCT02325219 | Risankizumab VS Placebo | 10.40714 | 3.933738 | 27.53326 | 1 |
| NCT02325219 | Secukinumab VS Placebo | 21.09091 | 5.797724 | 76.72433 | 1 |
| NCT02325219 | Tildrakizumab VS Placebo | 12.38519 | 4.890323 | 31.3666 | 1 |
| NCT02325219 | Ustekinumab VS Placebo | 13.2833 | 6.375378 | 27.67616 | 1 |
| NCT03364309 | Bimekizumab VS Placebo | 29.57576 | 14.44436 | 60.55828 | 1 |
| NCT03364309 | Brodalumab VS Placebo | 53.15556 | 15.46578 | 182.6946 | 1 |
| NCT03364309 | Guselkumab VS Placebo | 28.31394 | 20.75561 | 38.62469 | 1 |
| NCT03364309 | Ixekizumab VS Placebo | 41.00556 | 29.66364 | 56.68407 | 1 |
| NCT03364309 | Risankizumab VS Placebo | 10.40714 | 3.933738 | 27.53326 | 1 |
| NCT03364309 | Secukinumab VS Placebo | 21.09091 | 5.797724 | 76.72433 | 1 |
| NCT03364309 | Tildrakizumab VS Placebo | 12.38519 | 4.890323 | 31.3666 | 1 |
| NCT03364309 | Ustekinumab VS Placebo | 13.2833 | 6.375378 | 27.67616 | 1 |
| NCT03370133 | Brodalumab VS Placebo | 53.15556 | 15.46578 | 182.6946 | 1 |
| NCT03370133 | Guselkumab VS Placebo | 28.31394 | 20.75561 | 38.62468 | 1 |
| NCT03370133 | Ixekizumab VS Placebo | 42.33392 | 30.7526 | 58.27673 | 1 |
| NCT03370133 | Risankizumab VS Placebo | 10.40714 | 3.933738 | 27.53326 | 1 |
| NCT03370133 | Secukinumab VS Placebo | 21.09091 | 5.797724 | 76.72433 | 1 |
| NCT03370133 | Tildrakizumab VS Placebo | 12.38519 | 4.890323 | 31.3666 | 1 |
| NCT03897088 | Bimekizumab VS Placebo | 29.57576 | 14.44436 | 60.55828 | 1 |
| NCT03897088 | Brodalumab VS Placebo | 53.15556 | 15.46578 | 182.6946 | 1 |
| NCT03897088 | Guselkumab VS Placebo | 28.31394 | 20.75561 | 38.62469 | 1 |
| NCT03897088 | Ixekizumab VS Placebo | 42.33392 | 30.7526 | 58.27673 | 1 |
| NCT03897088 | Risankizumab VS Placebo | 10.40714 | 3.933738 | 27.53326 | 1 |
| NCT03897088 | Secukinumab VS Placebo | 21.09091 | 5.797724 | 76.72433 | 1 |
| NCT03897088 | Ustekinumab VS Placebo | 13.2833 | 6.375378 | 27.67616 | 1 |
| NCT05272150 | Bimekizumab VS Placebo | 29.57576 | 14.44436 | 60.55828 | 1 |
| NCT05272150 | Brodalumab VS Placebo | 53.15556 | 15.46578 | 182.6946 | 1 |
| NCT05272150 | Guselkumab VS Placebo | 29.24762 | 21.24144 | 40.27145 | 1 |
| NCT05272150 | Ixekizumab VS Placebo | 42.33392 | 30.7526 | 58.27673 | 1 |
| NCT05272150 | Risankizumab VS Placebo | 10.40714 | 3.933738 | 27.53326 | 1 |
| NCT05272150 | Secukinumab VS Placebo | 21.09091 | 5.797724 | 76.72433 | 1 |
| NCT05272150 | Tildrakizumab VS Placebo | 12.38519 | 4.890323 | 31.3666 | 1 |
| NCT05272150 | Ustekinumab VS Placebo | 13.2833 | 6.375378 | 27.67616 | 1 |
| NCT05969223 | Bimekizumab VS Placebo | 29.57576 | 14.44436 | 60.55828 | 1 |
| NCT05969223 | Brodalumab VS Placebo | 53.15556 | 15.46578 | 182.6946 | 1 |
| NCT05969223 | Guselkumab VS Placebo | 28.31394 | 20.75561 | 38.62469 | 1 |
| NCT05969223 | Ixekizumab VS Placebo | 42.33392 | 30.7526 | 58.27673 | 1 |
| NCT05969223 | Secukinumab VS Placebo | 21.09091 | 5.797724 | 76.72433 | 1 |
| NCT05969223 | Tildrakizumab VS Placebo | 12.38519 | 4.890323 | 31.3666 | 1 |
| NCT05969223 | Ustekinumab VS Placebo | 13.2833 | 6.375378 | 27.67616 | 1 |
| NCT06039189 | Bimekizumab VS Placebo | 29.57576 | 14.44436 | 60.55828 | 1 |
| NCT06039189 | Brodalumab VS Placebo | 53.15556 | 15.46578 | 182.6946 | 1 |
| NCT06039189 | Guselkumab VS Placebo | 31.32575 | 22.24284 | 44.11769 | 1 |
| NCT06039189 | Ixekizumab VS Placebo | 42.33392 | 30.7526 | 58.27673 | 1 |
| NCT06039189 | Risankizumab VS Placebo | 10.40714 | 3.933738 | 27.53326 | 1 |
| NCT06039189 | Secukinumab VS Placebo | 21.09091 | 5.797724 | 76.72433 | 1 |
| NCT06039189 | Tildrakizumab VS Placebo | 12.38519 | 4.890323 | 31.3666 | 1 |
| NCT06039189 | Ustekinumab VS Placebo | 13.2833 | 6.375378 | 27.67616 | 1 |
| UNCOVER_1_2_3 | Bimekizumab VS Placebo | 29.57576 | 14.44436 | 60.55828 | 1 |
| UNCOVER_1_2_3 | Brodalumab VS Placebo | 53.15556 | 15.46578 | 182.6946 | 1 |
| UNCOVER_1_2_3 | Guselkumab VS Placebo | 28.31394 | 20.75561 | 38.62469 | 1 |
| UNCOVER_1_2_3 | Ixekizumab VS Placebo | 142.45 | 19.32619 | 1049.974 | 1 |
| UNCOVER_1_2_3 | Risankizumab VS Placebo | 10.40714 | 3.933738 | 27.53326 | 1 |
| UNCOVER_1_2_3 | Secukinumab VS Placebo | 21.09091 | 5.797724 | 76.72433 | 1 |
| UNCOVER_1_2_3 | Tildrakizumab VS Placebo | 12.38519 | 4.890323 | 31.3666 | 1 |
| UNCOVER_1_2_3 | Ustekinumab VS Placebo | 13.2833 | 6.375378 | 27.67616 | 1 |

***Meta-Regression Analysis***

| **Table S24. Meta-Regression Analysis of Age for Complete Clearance in Scalp Psoriasis** | | | | | | | |
| --- | --- | --- | --- | --- | --- | --- | --- |
| Interventions | Covariates | Coefficient | Standard Error | Z-statistic | P>z | lower confidence interval | upper confidence interval |
| Brodalumab VS Bimekizumab |  |  |  |  |  |  |  |
|  | _cons | 0.5494237 | 2.762598 | 0.2 | 0.842 | -4.86517 | 5.964017 |
| Guselkumab VS Bimekizumab |  |  |  |  |  |  |  |
|  | Age | -0.1147214 | 6.662188 | -0.02 | 0.986 | -13.17237 | 12.94293 |
|  | _cons | 5.209697 | 307.1246 | 0.02 | 0.986 | -596.7434 | 607.1628 |
| Ixekizumab VS Bimekizumab |  |  |  |  |  |  |  |
|  | Age | -0.3325204 | 6.663846 | -0.05 | 0.96 | -13.39342 | 12.72838 |
|  | _cons | 15.41554 | 307.1975 | 0.05 | 0.96 | -586.6804 | 617.5115 |
| Placebo VS Bimekizumab |  |  |  |  |  |  |  |
|  | Age | -0.0921187 | 6.661584 | -0.01 | 0.989 | -13.14858 | 12.96435 |
|  | _cons | 0.8597209 | 307.099 | 0 | 0.998 | -601.0432 | 602.7627 |
| Risankizumab VS Bimekizumab |  |  |  |  |  |  |  |
|  | _cons | -1.072095 | 2.091652 | -0.51 | 0.608 | -5.171658 | 3.027469 |
| Secukinumab VS Bimekizumab |  |  |  |  |  |  |  |
|  | _cons | 0.0487893 | 27.98853 | 0 | 0.999 | -54.80772 | 54.9053 |
| Tildrakizumab VS Bimekizumab |  |  |  |  |  |  |  |
|  | _cons | -0.750696 | 8.68049 | -0.09 | 0.931 | -17.76414 | 16.26275 |
| Ustekinumab VS Bimekizumab |  |  |  |  |  |  |  |
|  | _cons | -0.8004469 | 0.2436256 | -3.29 | 0.001 | -1.277944 | -0.32295 |

| **Table S25. Meta-Regression Analysis of Previous Bio Therapy for Complete Clearance in Scalp Psoriasis** | | | | | | | |
| --- | --- | --- | --- | --- | --- | --- | --- |
| **Interventions** | **Covariates** | **Coefficient** | **Standard Error** | **Z-statistic** | **P>z** | **lower confidence interval** | **upper confidence interval** |
| Brodalumab VS Bimekizumab |  |  |  |  |  |  |  |
|  | _cons | 0.3172982 | 57.39562 | 0.01 | 0.996 | -112.176 | 112.8106 |
| Guselkumab VS Bimekizumab |  |  |  |  |  |  |  |
|  | Previous Bio Therapy | -0.0056204 | 7.551404 | 0 | 0.999 | -14.8061 | 14.79486 |
|  | _cons | 0.849623 | 294.5037 | 0 | 0.998 | -576.367 | 578.0662 |
| Ixekizumab VS Bimekizumab |  |  |  |  |  |  |  |
|  | Previous Bio Therapy | -0.0999132 | 7.551531 | -0.01 | 0.989 | -14.90064 | 14.70082 |
|  | _cons | 3.584598 | 294.5064 | 0.01 | 0.99 | -573.6374 | 580.8066 |
| Placebo VS Bimekizumab |  |  |  |  |  |  |  |
|  | Previous Bio Therapy | -0.035391 | 7.551382 | 0 | 0.996 | -14.83583 | 14.76505 |
|  | _cons | -2.006706 | 294.5036 | -0.01 | 0.995 | -579.2231 | 575.2097 |
| Risankizumab VS Bimekizumab |  |  |  |  |  |  |  |
|  | _cons | -0.9772178 | 14.36033 | -0.07 | 0.946 | -29.12296 | 27.16852 |
| Secukinumab VS Bimekizumab |  |  |  |  |  |  |  |
|  | _cons | 1.042136 | 294.5041 | 0 | 0.997 | -576.1753 | 578.2595 |
| Tildrakizumab VS Bimekizumab |  |  |  |  |  |  |  |
|  | _cons | 0.1806593 | 224.2762 | 0 | 0.999 | -439.3926 | 439.7539 |
| Ustekinumab VS Bimekizumab |  |  |  |  |  |  |  |
|  | _cons | -0.8004473 | 0.2436256 | -3.29 | 0.001 | -1.277945 | -0.32295 |

| **Table S26.** **Meta-Regression Analysis of** **Disease Duration for Complete Clearance in Scalp Psoriasis** | | | | | | | |
| --- | --- | --- | --- | --- | --- | --- | --- |
| **Interventions** | **Covariates** | **Coefficient** | **Standard Error** | **Z-statistic** | **P>z** | **lower confidence interval** | **upper confidence interval** |
| Brodalumab VS Bimekizumab |  |  |  |  |  |  |  |
|  | _cons | -0.157846 | 127.7042 | 0 | 0.999 | -250.4534 | 250.1377 |
| Guselkumab VS Bimekizumab |  |  |  |  |  |  |  |
|  | Disease Duration | -0.1611904 | 36.90813 | 0 | 0.997 | -72.4998 | 72.17742 |
|  | _cons | 2.718445 | 631.129 | 0 | 0.997 | -1234.272 | 1239.709 |
| Ixekizumab VS Bimekizumab |  |  |  |  |  |  |  |
|  | Disease Duration | -0.7518217 | 36.91008 | -0.02 | 0.984 | -73.09425 | 71.59061 |
|  | _cons | 14.04172 | 631.1699 | 0.02 | 0.982 | -1223.029 | 1251.112 |
| Placebo VS Bimekizumab |  |  |  |  |  |  |  |
|  | Disease Duration | -0.215063 | 36.90812 | -0.01 | 0.995 | -72.55365 | 72.12352 |
|  | _cons | 0.290626 | 631.1288 | 0 | 1 | -1236.699 | 1237.28 |
| Risankizumab VS Bimekizumab |  |  |  |  |  |  |  |
|  | _cons | -0.6788516 | 62.74672 | -0.01 | 0.991 | -123.6602 | 122.3025 |
| Secukinumab VS Bimekizumab |  |  |  |  |  |  |  |
|  | _cons | 0.1350297 | 81.2012 | 0 | 0.999 | -159.0164 | 159.2865 |
| Tildrakizumab VS Bimekizumab |  |  |  |  |  |  |  |
|  | _cons | -0.418818 | 77.50922 | -0.01 | 0.996 | -152.3341 | 151.4965 |
| Ustekinumab VS Bimekizumab |  |  |  |  |  |  |  |
|  | _cons | -0.8004469 | 0.2436256 | -3.29 | 0.001 | -1.277944 | -0.32295 |

| **Table S27. Meta-Regression Analysis of** **Baseline PASI for Complete Clearance in Scalp Psoriasis** | | | | | | | |
| --- | --- | --- | --- | --- | --- | --- | --- |
| **Interventions** | **Covariates** | **Coefficient** | **Standard Error** | **Z-statistic** | **P>z** | **lower confidence interval** | **upper confidence interval** |
| Brodalumab VS Bimekizumab |  |  |  |  |  |  |  |
|  | _cons | 0.8099552 | 62.23522 | 0.01 | 0.99 | -121.1688 | 122.7887 |
| Guselkumab VS Bimekizumab |  |  |  |  |  |  |  |
|  | Baseline PASI | -0.0640885 | 31.91353 | 0 | 0.998 | -62.61346 | 62.48528 |
|  | _cons | 1.441112 | 686.1406 | 0 | 0.998 | -1343.37 | 1346.252 |
| Ixekizumab VS Bimekizumab |  |  |  |  |  |  |  |
|  | Baseline PASI | 0.1329053 | 31.91406 | 0 | 0.997 | -62.41751 | 62.68332 |
|  | _cons | -2.208824 | 686.1507 | 0 | 0.997 | -1347.039 | 1342.622 |
| Placebo VS Bimekizumab |  |  |  |  |  |  |  |
|  | Baseline PASI | -0.1147191 | 31.91353 | 0 | 0.997 | -62.66409 | 62.43465 |
|  | _cons | -0.9205095 | 686.1406 | 0 | 0.999 | -1345.731 | 1343.89 |
| Risankizumab VS Bimekizumab |  |  |  |  |  |  |  |
|  | _cons | -0.0005348 | 290.4133 | 0 | 1 | -569.2001 | 569.1991 |
| Secukinumab VS Bimekizumab |  |  |  |  |  |  |  |
|  | _cons | 1.061444 | 389.3451 | 0 | 0.998 | -762.0409 | 764.1638 |
| Tildrakizumab VS Bimekizumab |  |  |  |  |  |  |  |
|  | _cons | -0.5607271 | 86.1682 | -0.01 | 0.995 | -169.4473 | 168.3258 |
| Ustekinumab VS Bimekizumab |  |  |  |  |  |  |  |
|  | _cons | -0.8004507 | 0.2436256 | -3.29 | 0.001 | -1.277948 | -0.32295 |

| **Table S28.** **Meta-Regression Analysis of** **Timepoint for Complete Clearance in Scalp Psoriasis** | | | | | | | |
| --- | --- | --- | --- | --- | --- | --- | --- |
| **Interventions** | **Covariates** | **Coefficient** | **Standard Error** | **Z-statistic** | **P>z** | **lower confidence interval** | **upper confidence interval** |
| Brodalumab VS Bimekizumab |  |  |  |  |  |  |  |
|  | _cons | 1.434005 | 234.1111 | 0.01 | 0.995 | -457.4153 | 460.2833 |
| Guselkumab VS Bimekizumab |  |  |  |  |  |  |  |
|  | _cons | -0.0435976 | 0.3984889 | -0.11 | 0.913 | -0.8246216 | 0.737426 |
| Ixekizumab VS Bimekizumab |  |  |  |  |  |  |  |
|  | _cons | 1.206371 | 234.1104 | 0.01 | 0.996 | -457.6416 | 460.0544 |
| Placebo VS Bimekizumab |  |  |  |  |  |  |  |
|  | Timepoint | -0.2119336 | 58.5276 | 0 | 0.997 | -114.9239 | 114.5001 |
|  | _cons | 0.0039862 | 936.4414 | 0 | 1 | -1835.388 | 1835.395 |
| Risankizumab VS Bimekizumab |  |  |  |  |  |  |  |
|  | _cons | -1.044459 | 0.6165085 | -1.69 | 0.09 | -2.252793 | 0.163876 |
| Secukinumab VS Bimekizumab |  |  |  |  |  |  |  |
|  | _cons | 0.5096251 | 234.1111 | 0 | 0.998 | -458.3398 | 459.359 |
| Tildrakizumab VS Bimekizumab |  |  |  |  |  |  |  |
|  | _cons | -0.8704504 | 0.5987194 | -1.45 | 0.146 | -2.043919 | 0.303018 |
| Ustekinumab VS Bimekizumab |  |  |  |  |  |  |  |
|  | _cons | -0.800447 | 0.2436256 | -3.29 | 0.001 | -1.277944 | -0.32295 |

***Supplementary Figures***

**Figure S1**. Risk of Bias Summary for Included Randomized Controlled Trials Using Cochrane RoB 2.0 Tool

**Figure S2**. Inconsistency Plots Assessing Loop-Specific Heterogeneity for Palmoplantar Psoriasis Network

**Figure S3**. Inconsistency Plots Assessing Loop-Specific Heterogeneity for Nail Psoriasis Network
